# Integration of food raw materials, food microbiology, and food additives: systematic research and comprehensive insights into sweet sorghum juice, *Clostridium tyrobutyricum* TGL-A236 and bio-butyric acid

**DOI:** 10.3389/fmicb.2024.1410968

**Published:** 2024-05-30

**Authors:** Mei-Han Liu, Xiang Zhou, Miao-Miao Zhang, Ya-Juan Wang, Bo Zhou, Nan Ding, Qing-Feng Wu, Cai-Rong Lei, Zi-Yi Dong, Jun-Le Ren, Jing-Ru Zhao, Cheng-Lin Jia, Jun Liu, Dong Lu, Hai-Yan Zhong

**Affiliations:** ^1^College of Food Science and Engineering, Central South University of Forestry and Technology, Changsha, China; ^2^Institute of Modern Physics, Chinese Academy of Sciences, Lanzhou, China; ^3^University of Chinese Academy of Sciences, Chinese Academy of Science, Beijing, China; ^4^Kejin Innovation Institute of Heavy Ion Beam Biological Industry, Baiyin, China; ^5^Gansu Key Laboratory of Microbial Resources Exploitation and Application, Lanzhou, China; ^6^Hunan Key Laboratory of Forestry Edible Resources Safety and Processing, Changsha, Hunan, China

**Keywords:** sweet sorghum juice, *Clostridium tyrobutyricum* TGL-A236, bio-butyric acid, qRT-PCR, pathway and process enrichment, protein homology modeling, protein cavity

## Abstract

**Introduction:**

Sweet sorghum juice is a typical production feedstock for natural, eco-friendly sweeteners and beverages. *Clostridium tyrobutyricum* is one of the widely used microorganisms in the food industry, and its principal product, bio-butyric acid is an important food additive. There are no published reports of *Clostridium tyrobutyricum* producing butyric acid using SSJ as the sole substrate without adding exogenous substances, which could reach a food-additive grade. This study focuses on tailoring a cost-effective, safe, and sustainable process and strategy for their production and application.

**Methods:**

This study modeled the enzymolysis of non-reducing sugars via the first/second-order kinetics and added food-grade diatomite to the hydrolysate. Qualitative and quantitative analysis were performed using high-performance liquid chromatography, gas chromatography-mass spectrometer, full-scale laser diffraction method, ultra-performance liquid chromatography–tandem mass spectrometry, the cell double-staining assay, transmission electron microscopy, and Oxford nanopore technology sequencing. Quantitative real-time polymerase chain reaction, pathway and process enrichment analysis, and homology modeling were conducted for mutant genes.

**Results:**

The treated sweet sorghum juice showed promising results, containing 70.60 g/L glucose and 63.09 g/L fructose, with a sucrose hydrolysis rate of 98.29% and a minimal sucrose loss rate of 0.87%. Furthermore, 99.62% of the colloidal particles and 82.13% of the starch particles were removed, and the concentrations of hazardous substances were effectively reduced. A food microorganism *Clostridium tyrobutyricum* TGL-A236 with deep utilization value was developed, which showed superior performance by converting 30.65% glucose and 37.22% fructose to 24.1364 g/L bio-butyric acid in a treated sweet sorghum juice (1:1 dilution) fermentation broth. This titer was 2.12 times higher than that of the original strain, with a butyric acid selectivity of 86.36%. Finally, the Genome atlas view, Gene Ontology (GO), Kyoto Encyclopedia of Genes and Genomes (KEGG), and evolutionary genealogy of genes: Non-supervised Orthologous (eggNOG) functional annotations, three-dimensional structure and protein cavity prediction of five non-synonymous variant genes were obtained.

**Conclusion:**

This study not only includes a systematic process flow and in-depth elucidation of relevant mechanisms but also provides a new strategy for green processing of food raw materials, improving food microbial performance, and ensuring the safe production of food additives.

## 1 Introduction

To attain the 1.5°C global warming mitigation target established by the 2016 Paris Agreement and advance the shared goal of global carbon neutrality, the pursuit of clean alternative energy sources is vital for global climate governance and sustainable economic and environmental development (Jiang et al., 2022). Sweet sorghum, a form of green biomass, is one of the best alternative energy sources. The Food and Agriculture Organization of the United Nations estimated global sweet sorghum biomass production in 2021 was as high as 14,994 kg/ha. Nevertheless, high output does not necessarily mean high value. Ghana, for instance, currently sells sweet sorghum for only $59.4 per metric ton, despite its notable sweet sorghum production (FAOSTAT, 2022; Tulashie et al., 2023). The surplus of green biomass poses challenges, such as devaluation of selling prices, overcapacity, and storage difficulties. The juice yield from sweet sorghum stalks is generally below 55%, containing high amounts of nutrients, including non-structural soluble sugars, such as sucrose (53–85%), glucose (9–33%), and fructose (6–21%), as well as starch, amino acids, and inorganic salts. Additionally, secondary products such as aconitic acid, organic acids, and phenolic substances are also accumulated (Uchimiya et al., 2017; Appiah-Nkansah et al., 2019; Husiatynska et al., 2021). Efforts to increase the value of sweet sorghum have been ongoing since the 1970s, focusing on converting sweet sorghum juice (SSJ) into biofuels or bioproducts such as ethanol, methane, hydrogen, and L-lactic acid. Utilizing excess sweet sorghum to produce food additives, like bio-butyric acid, presents an opportunity for further optimizing and upgrading the industry chain (Appiah-Nkansah et al., 2019; Zhu et al., 2023).

Butyric acid, a typical short-chain fatty acid, has been approved by the U.S. Food and Drug Administration and many other countries as a food flavoring agent. Butyric acid and its derivatives have diverse applications in the food, pharmaceutical, and energy industries. They serve as intermediate compounds for synthesizing γ-aminobutyric acid (GABA), which is a biologically active substance permitted by the European Food Safety Authority (EFSA) to be added to food or beverages, up to an intake of 550 mg/d. Moreover, they are employed to treat strokes and regulate neurological disorders such as Parkinson’s and Alzheimer’s diseases. Butyric acid is also used as a histone deacetylase inhibitor for treating intestinal inflammation, cardiovascular disease, and cancer. Butyric acid can further serve as a substrate to produce biofuel butanol through hydrogen-catalyzed reduction (EFSA Panel on Dietetic Products, Nutrition and Allergies, 2009; Alimentarius, 2015; Sarasa et al., 2020; Oketch-Rabah et al., 2021). Microbially synthesized bio-butyric acid is gaining traction due to consumer preferences for natural products and the global emphasis on environmental sustainability, particularly the concept of carbon neutrality. Sweet sorghum, recognized for its safety as a non-food crop, presents an opportunity to enhance its value. The stalk juice of sweet sorghum is abundant in free sugars and trace elements, making it an ideal substrate for microbial fermentation. Utilizing SSJ to produce high-value bio-butyric acid aligns with both consumer preferences and global environmental goals (Dar et al., 2018).

The Food and Agriculture Organization (FAO) of the United Nations and the World Health Organization jointly convened the 52nd Expert Committee on Food Additives in Rome, noting that Clostridium is a normal intestinal bacterium in humans. Among the butyric acid-producing strains, *Clostridium tyrobutyricum* is recognized as one of the most promising cellular factories for butyric acid synthesis because of its high butyric acid titer (Bao et al., 2020). Unfortunately, there are technical bottlenecks that must be overcome to utilize SSJ as a substrate for butyric acid fermentation: (1) Colloidal particles and insoluble starch in SSJ increase liquid viscosity, hampering the clarification and impurity removal processes. This interference leads to cell aggregation and attachment to colloidal particles, diminishing fermentation efficiency (Eggleston et al., 2016); (2) sweet sorghum stalks typically contain 2.21–5.77 mg/g phenolic compounds, and dissolution of these compounds in the juice can negatively affect the growth and metabolism of microorganisms (Chen et al., 2021, 2022; Bakari et al., 2023); (3) the high sucrose content in SSJ serves as the main carbon substrate. However, *C. tyrobutyricum* cannot utilize sucrose directly; it must undergo hydrolysis to convert sucrose into fermentable sugars for use as a fermentation substrate. Although some chemical methods have been investigated to remove colloidal and bacteriostatic components and achieve complete sucrose hydrolysis, these methods are not environmentally friendly. This usually results in a more complex composition of SSJ, adding an additional economic cost to the removal of impurities and food safety risks (Costa et al., 2015). Additionally, the industrial application of microbial fermentation for butyric acid faces many challenges, such as limited butyric acid tolerance in wild-type *C. tyrobutyricum*, byproduct generation and isolation, and product inhibition. To address these challenges, heavy-ion beam (HIB) irradiation mutagenesis emerges as a promising approach. Its high mutation stability and biological effects make HIB irradiation a widely used method in various industries, including food, medicine, agriculture, energy, chemicals, and environmental protection. The application of HIB irradiation as a means of selecting and breeding *C. tyrobutyricum* holds promise for enhancing butyric acid production, offering both feasibility and mutagenicity benefits (Zhou et al., 2016; Guo et al., 2019; Gao et al., 2021; Shao et al., 2022; Niwano, 2023).

Therefore, this study aims to develop an efficient and sustainable SSJ treatment process to achieve sucrose hydrolysis, remove colloidal particles and impurities, significantly reduce toxic substance concentrations, and obtain a fermentation substrate that is better adapted to the growth and metabolism of *Clostridium* spp. HIB irradiation was selected as the mutagenesis method for *C. tyrobutyricum*, through a combination with adaptive laboratory evolution, leading to the breeding of a remarkable mutant strain, *C. tyrobutyricum* TGL-A236. The fermentation performance of this mutant strain was rigorously evaluated using fermentation kinetics. Subsequently, an in-depth analysis was conducted at the gene level and through protein structure prediction to elucidate the mechanisms underlying growth adaptability in SSJ, and the increased titer of butyric acid in the mutant strain. These results not only provide a new direction for the industrial application of sweet sorghum juice as a food raw material and the extension of its industrial chain but also lay the groundwork for enhancing the butyric acid synthesis flux in *C. tyrobutyricum*, offering valuable insights for further industrial applications of food additive butyric acid.

## 2 Materials and methods

### 2.1 Green pretreatment of SSJ

#### 2.1.1 Raw material extraction and separation

Sweet sorghum was harvested in October 2022 by the Kejian Heavy-ion Beam Bio-Industrial Innovation Research Institute located in Baiyin City, Gansu Province, China. Sweet sorghum stalks were squeezed using a three-roller mill, and the resulting juice was centrifuged at 4,000 g/min for 10 min to produce a supernatant, which was subsequently collected. The pH was regulated to 5.0 with 1 M phosphoric acid solution and autoclaved for 20 min at 115°C before being frozen at −20°C for preservation.

#### 2.1.2 Catalytic hydrolysis of non-reducing sugars

The content of non-reducing sugars (sucrose) in SSJ reaches 99.82%, making other trace non-reducing sugars negligible (Andrzejewski et al., 2013b). Invertase (CAS NO: 9001-57-4, ≥200 units/mg solid; HRBS-M019, Hongrunbaoshun, Beijing, China) was added to the SSJ at a concentration of 0.01% (w/v) or 20 units/mL. Additionally, a 0.005% (w/v) concentration, equivalent to 10 units/mL, was added to a 1:1 dilution of SSJ. The hydrolysis reaction was performed in a shaker at 42°C and 150 rpm for 24 h. Samples were collected at 0, 6, 12, 18, and 24 h. The sugar composition of the hydrolysate was determined, and the degree of sucrose hydrolysis and conversion was analyzed using high-performance liquid chromatography (HPLC).

#### 2.1.3 Removal of colloids and impurities

To the hydrolysate, 5% (w/v) diatomite (CAS NO: 61870–53-2, Damao, Tianjing, China) was incorporated and subjected to shaking at 42°C and 150 rpm for 4 h. The pH of the hydrolysate was then adjusted to a range of 6.2–6.5 using a 5 M NaOH solution. The hydrolysate was allowed to settle for 30 min and then centrifuged at 4,000 *g*/min for 10 min. The supernatant was subsequently collected and stored in a refrigerator at −20°C.

#### 2.1.4 Kinetic model for sucrose hydrolysis

The sucrose hydrolysis reaction was assessed using first-order and second-order kinetic models, with the enzymatic reaction rate quantified as the increment in invertose released during sucrose hydrolysis per unit time (Gonzàlez-Tello et al., 1994; de Souza Soares et al., 2019).

The first-order kinetic model is shown in Eq. (1):


(1) $$-\frac{d\left(C_{\infty }-C_{t}\right)}{dt}=K_{reaction}\cdot \left(C_{\infty }-C_{t}\right)$$

Eq. (2) was obtained by integrating Eq. (1):


(2) $$\ln\left(C_{\infty }-C_{t}\right)=-K_{reaction}+\ln C_{\infty }$$

Converting Eq. (2) yields Eq. (3):


(3) $$C_{t}=C_{\infty }-\exp\left(\ln C_{\infty }-K_{reaction}\cdot t\right)$$

The second-order kinetic model is shown in Eq. (4):


(4) $$\frac{dC_{t}}{dt}=K_{reaction}\cdot {\left(C_{\infty }-C_{t}\right)}^{2}$$

Integrating Eq. (4) results in Eq. (5):


(5) $$C_{t}=\frac{C_{\infty }{\;}^{2}\cdot K_{reaction}\cdot t}{1+C_{\infty }\cdot K_{reaction}\cdot t}$$

where *t* denotes the enzymatic reaction time (h); *C_t_* signifies the increase in invertose (i.e., fructose and glucose obtained by the hydrolysis of sucrose) concentration at time *t* (g/L); *C_∞_* is the increase in the final concentration of invertose (g/L); *K_reaction_* is the reaction hydrolysis rate constant (h^−1^) for a given set of conditions such as temperature and pH.

### 2.2 Strain, medium, and adaptive laboratory evolution

#### 2.2.1 Strain

The original strain (*C. tyrobutyricum* strain Cirm BIA 2237) was provided by the Institute of Modern Physics, Chinese Academy of Sciences, and stored in the Biophysics Department of the Biomedical Center.

#### 2.2.2 Medium

Reinforced *Clostridium* medium (RCM) composition: peptone: 10.0 g/L; beef meal: 10.0 g/L; yeast extract: 3.0 g/L; glucose: 5.0 g/L; soluble starch: 1.0 g/L; sodium chloride: 5.0 g/L; sodium acetate: 3.0 g/L; L-cysteine hydrochloride: 0.5 g/L.

For the adaptive laboratory evolution/fermentation medium, yeast extract (3.0 g/L) was used as nitrogen source. Depending on the experimental design, the carbon source was selected including untreated SSJ, pretreated SSJ or 1:1 dilution of SSJ (serving as a medium solvent instead of water).

Solid culture was facilitated by the addition of 20 g/L agar, and all media underwent autoclaving at 115°C for 20 min before use.

#### 2.2.3 Adaptive laboratory evolution of *Clostridium tyrobutyricum*

Following the methodology outlined in Wang et al. (2023), the original strain underwent successive passaging from plate to liquid culture using an adaptive evolution medium. This process was reiterated until the 4th generation to obtain the domesticated strain. The GenBank Accession Number of the domesticated strain (*Clostridium tyrobutyricum* TGL) was SUB14362386 Clostridium PP593753.

### 2.3 HIB irradiation treatment and screening of high-yielding butyric acid mutant strains

#### 2.3.1 HIB irradiation treatment of *Clostridium tyrobutyricum*

The ^12^C^6+^ HIB was provided by the Heavy-Ion Research Facility of Lanzhou at the Institute of Modern Physics, Chinese Academy of Sciences, with an irradiation energy of 80 MeV/u, a linear energy transfer of 40–50 KeV/μm, an absorbed dose rate set at 20 Gy/min, and a beam spot diameter of 40 mm.

The *C. tyrobutyricum* strain Cirm BIA 2237 was cultured in RCM medium and incubated anaerobically at 37°C until reaching an OD_600_ of 0.8–1.0. Next, 1 mL of the logarithmic phase cell suspension was dispensed and sealed in 35 mm sterile petri dishes. Cell suspensions of the original strain were exposed to varying doses of high-energy ^12^C^6+^ ion beams at 0, 60, 120, 180, 240, 300, and 360 Gy. Three sets of samples were prepared in parallel for each dose.

#### 2.3.2 Strain lethality assay

After irradiation, an appropriate amount of bacterial solution was collected for dilution, inoculated onto RCM plates at 100 μL/plate, and incubated at 37°C in an anaerobic environment for 72 h. The number of colonies grown on each plate was counted, and the viable count per milliliter of broth (cfu/mL) was calculated. A pre-irradiation (irradiation dose of 0 Gy) plate was used as the control, and the lethality of the strains after treatment with each dose was calculated using Eq. (6):


(6) $$\begin{array}{l} \mathrm{Lethality rate}=\\ \frac{viablecountbeforeirradiation\;-\;viablecountafterirradiation}{viablecountbeforeirradiation}\times 100\% \end{array}$$

#### 2.3.3 Markov Chain Monte Carlo (MCMC) modeling and fitting

The model was constructed as previously described by Zhou et al. (2016). The model parameters were estimated by combining the MCMC method. The optimal irradiation dose for the high-yielding butyric acid mutant was determined based on the model predictions.

#### 2.3.4 Screening of high-yielding butyric acid mutant strains

The strains that underwent irradiation and had a predicted lethality rate of 80–90% according to the MCMC model were studied (Matuo et al., 2006). These strains underwent adaptive laboratory evolution in a solid–liquid culture mode until the 4th generation, using the medium mentioned in Section 2.2.3. The highest butyric acid-producing capacity (lowest pH) was used as the screening condition for each generation. Bio-acid production in the broth was determined using gas chromatography–mass spectrometry (GC–MS). The pH was measured using a pH meter (PB-10, Sartorius, Germany), cell density was determined by measuring the absorbance at 600 nm using a microplate spectrophotometer (Epoch, BioTek, USA), and gas production was measured cumulatively using 50 mL sterile syringes.

### 2.4 Cell double-staining assay

The original, domesticated, and mutant strains were incubated in RCM medium at 37°C and activated until the broth’s OD_600_ reached approximately 0.8. Subsequently, each of the three broths was inoculated with a 5% (v/v) inoculum into anaerobic flasks containing either sterile untreated SSJ or treated SSJ.

The cultures were maintained anaerobically at 37°C for 24 h. Samples were collected at intervals of 0, 6, 12, 18, and 24 h for the cell double-staining assays. Staining was performed using the Annexin V-FITC/PI Apoptosis Detection Kit (Cat. No. CA 1020, China). A 1 mL aliquot of the cell suspension was centrifuged at 2000 *g*/min for 5 min to remove the supernatant. The cell pellet was then resuspended in 1 mL of pre-cooled 1X PBS at 4°C, followed by centrifugation to collect the precipitate. The concentration was adjusted to 1–5 × 10^6^ mL^−1^ in 1X Binding Buffer. In a 1.5 mL centrifuge tube, 100 μL of the cell suspension was mixed with 5 μL of Annexin V/FITC dye and incubated for 5 min at room temperature, avoiding light exposure. Subsequently, 5 μL of PI dye and 200 μL of 1X PBS were added and mixed well, and the apoptosis rate was detected immediately using flow cytometry (Amnis^®^ Flowsight^®^, Milli-Q, USA). FITC showed green fluorescence in the Ch_02 channel, and the PI-DNA complex showed red fluorescence in the Ch_04 channel. Subsequently, 10,050 ± 50 cells were selected for each sample, and the data were analyzed using IDEAS Application 6.0 software (Amnis Inc., USA).

### 2.5 Biofermentation assay

Single colonies of the original strain and mutant strain grown in the adaptive laboratory evolution plates were picked and inoculated into the fermentation broth. The OD_600_ values of the two bacterial suspensions were adjusted to 0.7 after anaerobic incubation at 37°C for 20–24 h. The inoculum was transferred to an anaerobic fermentation flask containing 200 mL of fermentation medium at a 20% (v/v) inoculum volume, and the flask was sealed after passing through sterile nitrogen gas. The fermentation flasks were incubated in a 37°C incubator for 12 h and transferred to a thermostatic shaker at 37°C and 150 rpm for 120 h. The fermentation medium was supplemented with 100 mL of fermentation medium and incubated for up to 36 h. During fermentation, a sterile syringe was employed every 2–6 h to release gas, preventing bottle explosion from excessive air pressure generated by fermentation gas production. Samples were collected at 0, 24, 48, 72, 96, and 120 h of fermentation, and the OD_600_, gas production, glucose and fructose contents, and butyric and acetic acid titers of the fermentation broth were measured.

### 2.6 Transmission electron microscopy

The original and mutant strains were fermented in untreated/treated SSJ medium for 18 h. Post-fermentation, 2 mL of each broth was pipetted, followed by centrifugation at 6,000 g/min for 5 min. The resulting bacterial precipitate was collected after discarding the medium. The cells were resuspended twice using 1X PBS buffer, fixed with 2.5% (v/v) glutaraldehyde solution for 2 h at 20–25°C, and stored at 4°C. The solution was resuspended three times with 0.1 M phosphate buffer PB (pH 7.4) and rinsed for 3 min each time. The solution was heated and dissolved to prepare a 1% (w/v) agarose solution. A slightly cooled solution was added to the EP tubes, and the suspension packages were picked and wrapped in agarose before solidification. Osmium was fixed in 1% osmium acid prepared in 0.1 M phosphate buffer PB (pH 7.4) for 2 h at room temperature in the dark and rinsed three times with 0.1 M phosphate buffer PB (pH 7.4) for 15 min each time. For dehydration, a sequential process was employed using 30, 50, 70, 80, 95, and 100% (v/v) ethanol solutions for 20 min and 100% acetone twice for 15 min each. Subsequently, a mixture of acetone and EMBed 812 in a ratio of 1:1 was applied for 2–4 h at 37°C, followed by a ratio of acetone to EMBed 812 at 1:2 overnight at 37°C, and finally, pure EMBed 812 for 5–8 h at 37°C. Pure EMBed 812 was poured into embedding models, and tissues were inserted into the resin, with the entire setup kept in a 37°C oven overnight. The embedding models, now containing resin and samples, were then transferred to a 60°C oven to polymerize for 48 h and resin blocks were cut using an ultra microtome (Leica, UC7, Germany). After staining with a 2% (v/v) uranyl acetate-saturated ethanol solution and a 2.6% (v/v) lead citrate solution, the samples were observed under a transmission electron microscope (Hitachi, HT7800, Japan).

### 2.7 Physical and chemical analysis

#### 2.7.1 Determination of particle size and distribution using the full-scale laser diffraction method

The particle size distribution of untreated and treated SSJ was measured using a wet method fully automatic laser particle size analyzer (NKY5100-H, SDNKT, Shandong, China). The test parameters were as follows: the refractive index of the sample was 1.61–0.1i, the refractive index of the medium was 1.33, the dispersing medium was water, the range of tested particle sizes was 0.1–800 μm, the ultrasonic power was 50 W, and the circulating flow rate was 1 L/min. After completing the water intake, bubble evacuation, and background collection procedures, the machine was preheated for 30 min before testing 50 mL of untreated and treated SSJ, respectively.

#### 2.7.2 Determination of sugar composition using high performance liquid chromatography (HPLC)

HPLC (Bruker, Germany) was used to determine the main sugar components in SSJ with the following specifications: (1) Chromatographic column: Zafex Carbohydrate ES (250 × 4.6 mm, 5 μm). (2) Chromatography conditions: Mobile phase A-25% ultrapure water, mobile phase B-75% acetonitrile; flow speed: 1.0 mL/min; column temperature: 40°C; refractive index (RI) detector temperature: 30°C; injection volume: 15.00 μL; running time: 25 min. (3) Sample preparation and detection: fructose, glucose, and sucrose were oven-dried at 90°C until a constant weight was achieved. A 10 g/L standard mother solution of fructose, glucose, and sucrose was prepared. Standards of fructose, glucose, and sucrose were diluted to concentrations of 1, 2, 4, 6, 8, and 10 g/L. Untreated/treated samples were diluted with ultrapure water for 10X as the test samples. All the standards and samples were passed through 0.22 μm aqueous filters and then detected by the machine.

#### 2.7.3 Determination of hazardous substances using ultra-performance liquid chromatography–tandem mass spectrometry (UPLC-MS/MS)

(1) Sample pretreatment: Samples were extracted with a mixture of methanol, water, and formic acid in a 15:4:1 (v:v:v) ratio. After extraction, the samples were concentrated through centrifugation at 12,000 g/min at 4°C, dried using nitrogen blowing, and redissolved with 100 μL of 80% acetonitrile solution. The reconstituted samples were then filtered through 0.22 μm PTFE filters for online assessment. (2) Chromatography conditions: A Thermo Q-Exactive HF liquid chromatography-mass spectrometer with an Agilent C18 column (2.1 mm × 100 mm, 3 μm) was utilized. The column temperature was set at 35°C, with a flow rate of 0.3 mL/min and an injection volume of 10.00 μL. The mobile phase comprised a gradient elution of 0.1% formic acid solution (A), and 100% acetonitrile (B). (3) Mass Spectrometry Conditions: Positive/negative ion mode was used with the following parameters: heater temperature at 325°C, sheath gas flow rate of 45 arb, auxiliary gas flow rate of 15 arb, purging gas flow rate of 1 arb, electrospray voltage at 3.5 KV, capillary temperature at 330°C, and S-Lens RF Level at 55%. The scanning mode deployed comprised a one-stage full scan (full scan, m/z 100–1,500) and a data-dependent two-stage mass spectrometry scan (dd-MS2, TopN = 10). Resolutions were set at 120,000 for primary MS and 60,000 for secondary MS. Implemented high-energy collision dissociation as the collision mode.

#### 2.7.4 Determination of bio-acids using gas chromatography coupled with mass spectrometry (GC–MS)

(1) Sample preparation: An appropriate volume (40–100 μL) of fermentation broth was added to 2 mL of water (1,3 aqueous phosphoric acid). The mixture was vortexed for 2 min, followed by the addition of 2 mL of ether. After 10 min, the solution was centrifuged at 4,000 g/min for 20 min in an ice water bath, and the ether phase was removed. The ether extraction process was repeated, and the two extracts were combined and volatilized to 2 mL for GC–MS injection analysis. (2) Chromatographic conditions: An Agilent 7890B-7000D gas chromatography-mass spectrometer (GC–MS) with an HP-INNOWAX column (25 mm × 0.20 mm × 0.40 μm) was used. The column temperature initiated at 100°C, held for 5 min, increased to 150°C at 5°C/min, further increased to 240°C at 30°C/min, and maintained for 30 min. The inlet temperature was set at 240°C, with a carrier gas flow rate of 1.0 mL/min and no split ratio. (3) Mass spectrometry conditions: Operated in a single-ion scanning mode with quantitative ions at 60 and 73. The ion source temperature and transmission line temperature were set at 200°C and 250°C, respectively. Utilized an electron impact ionization mode with a bombardment voltage of 70 eV.

### 2.8 Oxford nanopore technologies (ONT) whole-genome sequencing and functional annotation of mutant strains

The original strain (*C. tyrobutyricum* Cirm BIA 2237) and mutant strain (*C. tyrobutyricum* TGL-A236) were diluted and spread onto RCM plates for anaerobic incubation for 2–3 days at 37°C. Well-growing colonies were individually selected, inoculated into RCM broth, and incubated at 37°C for 18–24 h until reaching an OD_600_ of 0.8 or above. The bacterial precipitate was collected by aspirating 1 mL of each of the original and mutant strains and centrifuging at 10,000 g/min for 2 min. After resuspending the bacteria twice with 1X PBS buffer, they were snap-frozen in liquid nitrogen for 0.5–1 h and subsequently stored at −80°C overnight.

Genomic DNA extraction, library construction, quality testing, sequencing, and information analysis were conducted by Biomarker Technologies Company in Beijing, China, following the standard protocol of ONT. The experimental steps included: (1) Total DNA extraction and quality assessment using Nanodrop, Qubit, and 0.35% agarose gel electrophoresis; (2) recovery of large DNA fragments using the BluePippin fully automated nucleic acid recovery system; (3) library construction using the SQK-LSK109 Ligation Kit, involving DNA damage/end repair, ligation, and magnetic bead purification; (4) library quantification using Qubit; (5) library quality testing and sequencing on the OXFORD_NANOPORE Promethion platform. Raw reads obtained from sequencing underwent quality assessment and filtering. The filtered reads were assembled using Canu v1.5 software, followed by circularization and assembly of the genome using Circlator v1.5.5 software. The whole-genome sequence data of the mutant strain *C. tyrobutyricum* TGL-A236 have been deposited at the China National Center for Bioinformation/Beijing Institute of Genomics, Chinese Academy of Sciences, under Genome Sequence Archive No. CRA013003. The genome of the mutant strain was functionally annotated in GO^1^, KEGG^2^, and eggNOG^3^ databases by BLAST alignment.

### 2.9 Quantitative real-time PCR analysis of mutant genes

**Figure 1:**
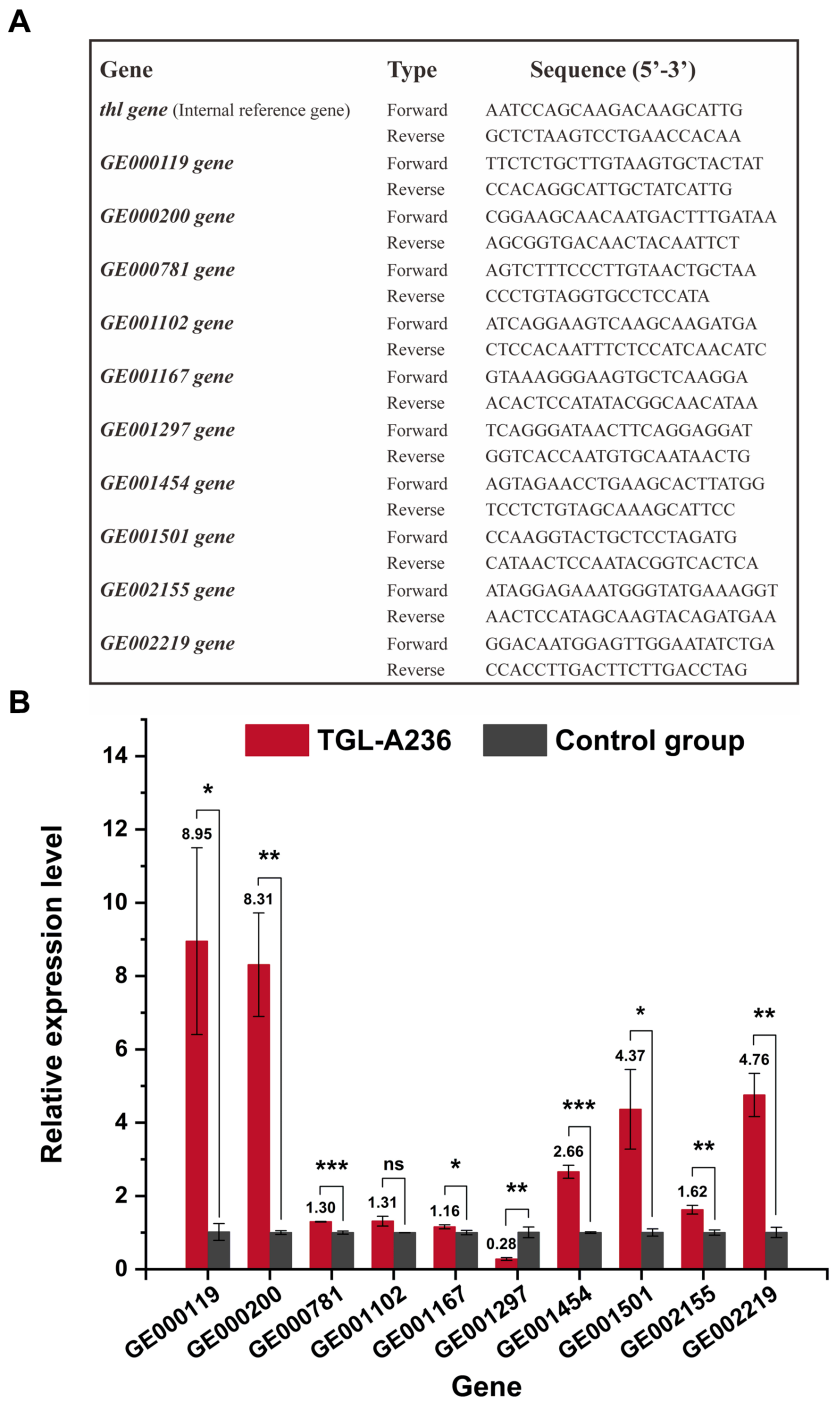
Quantitative real-time polymerase chain reaction (qRT-PCR) results of *C. tyrobutyricum* TGL-A236 mutant genes. **(A)** Primers used to detect *C. tyrobutyricum* TGL-A236 in the real-time quantitative PCR analysis. **(B)** The relative expression of mutant genes was measured after 12 h of incubation in the fermentation medium. Black columns represent *C. tyrobutyricum* TGL-A236 strain, while gray columns represent *C. tyrobutyricum* Cirm BIA 2237 strain (control group). The amplification and melt curve plots are shown in Supplementary Figures S3–S13. *means a significance level of *p* < 0.05, **means a high significance level of *p* < 0.01, and ***means an extreme significance level of *p* < 0.001.

The relative expression levels of mutant genes were investigated using qRT-PCR. *C. tyrobutyricum* TGL-A236 was inoculated in a fermentation medium and cultured at 37°C for 12 h. Strain cells (1 mL) were collected via centrifugation (4°C, 10,000 g/min for 5 min). The bacteria total RNA isolation kit (NO. B518625, Sangon Biotech, China) was used for the extraction of total RNA. The FastKing gDNA dispelling RT SuperMix kit (KR118, Tiangen, China) was used for reverse transcription. SuperReal PreMix plus (SYBR Green) kit (FP205, Tiangen, China) and QuantStudioTM5 Real-Time PCR instrument (Thermo Fisher, USA) were used for qRT-PCR analysis. All specific primers for mutant genes were designed using the NCBI Primer-BLAST tool (listed in Figure 1A). Thiolase gene (*thl*) was selected as the internal reference/endogenous control gene, and the 2^−ΔΔCt^ method was used to process the qRT-PCR data (Fu et al., 2022). Relative gene expression of *C. tyrobutyricum* Cirm BIA 2237 under the same culture conditions was used as a control group.

### 2.10 Pathway and process enrichment analysis of mutant genes

The sequences of the top five mutant genes with the highest relative expression levels in the qRT-PCR experiments were selected. The Gene Ontology (GO) function enrichment analysis was, respectively, performed for these 5 differentially-expressed genes with the following ontology sources: including Biological Processes, Molecular function, KEGG pathways, Reactome Gene Sets, Canonical Pathways, CORUM, WikiPathways, and PANTHER pathways. The ontology sources of these homologous genes were analyzed for pathway and process enrichment using Cytoscape (v3.10.0) software.

### 2.11 Homology modeling of mutant gene-encoded proteins

The clean reads obtained were compared with the reference genome sequence using Mummer (v.4.0.0beta) to identify mutant genes, including single nucleotide polymorphisms (SNPs), insertion deletions (Indels), and other variation sites. Additionally, gene function annotation was performed. For non-synonymous mutant genes, protein homology modeling was conducted. The minimum ORF length of the nucleic acid sequence was set to 75 nt, and only “ATG” was considered as the start codon for translating the amino acid sequence within the coding sequence region. The resulting amino acid sequence was used to determine the largest sequence identity in the China Protein Structure Database and UniProtKB Database, and the MolProbity results were analyzed for structural integrity and quality.

### 2.12 Statistical methods and data analysis

Data were analyzed using IBM SPSS Statistics 26 software (SPSS Inc., USA). One-way analysis of variance was used to compare multiple groups. Pairwise comparisons were conducted using the Least Significant Difference method and Turkey’s test, with a significance level of *p* < 0.05 and a high significance level of *p* < 0.01.

## 3 Results

### 3.1 Comprehensive evaluation of green treatment processes for SSJ

#### 3.1.1 Hydrolysis and conversion of non-reducing sugars in SSJ before/after treatment

**Figure 2:**
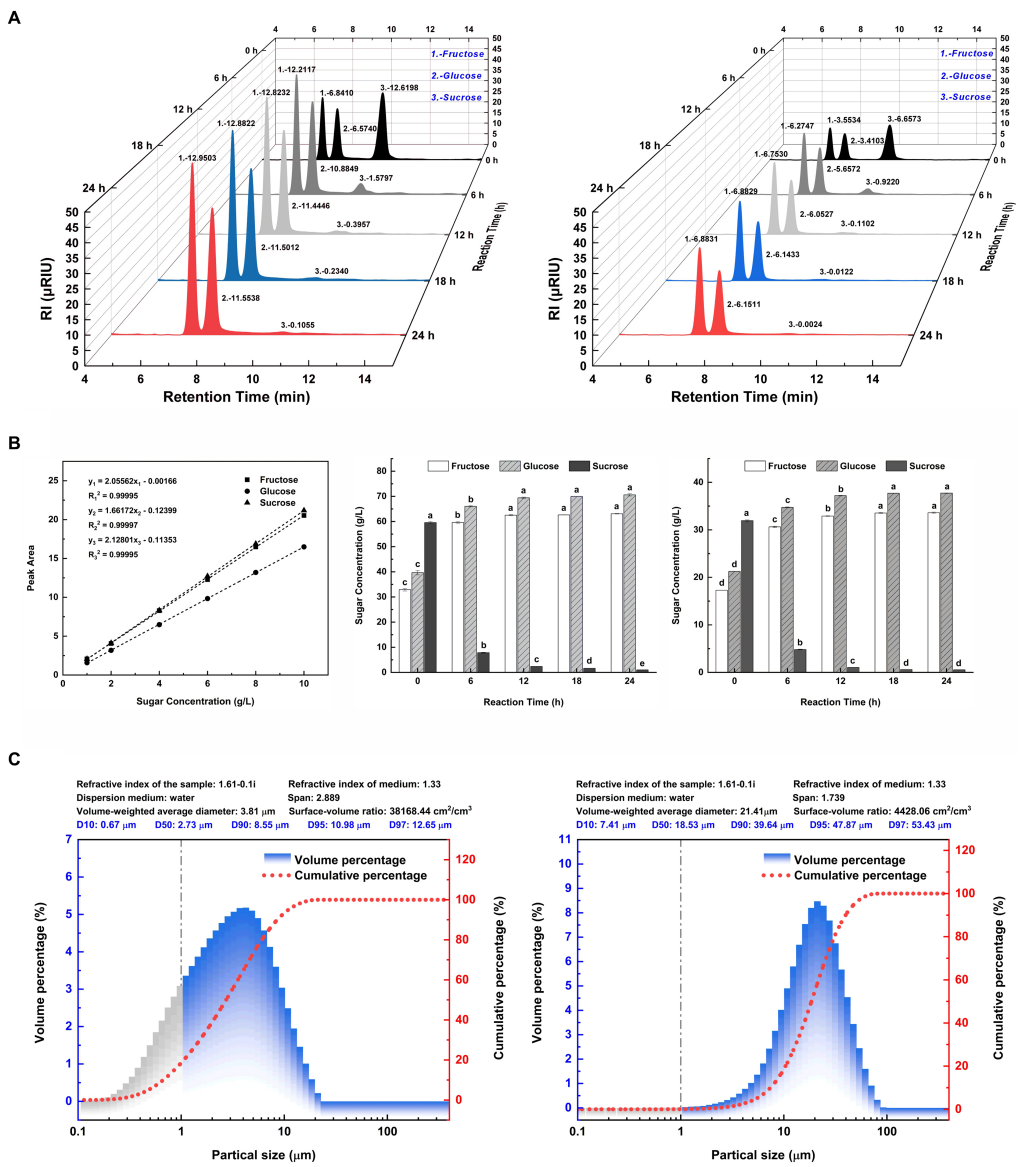
Major sugar components and particle size distribution of SSJ before and after green treatment. **(A)** (left) A 3D waterfall plot of peaks from liquid chromatography of SSJ treated with 20 units/mL invertase for 24 h. **(A)** (right) A 3D waterfall plot from a 1:1 dilution of SSJ treated with 10 units/mL invertase for the same duration. The *X*-axis represents the retention time (min) of sample components (peak No. 1: fructose, peak No. 2: glucose, peak No. 3: sucrose). The *Y*-axis denotes the hydrolysis reaction time (h) with black, dark gray, light gray, blue, and red peaks indicating 0, 6, 12, 18, and 24 h enzymatic reactions, respectively. The *Z*-axis shows the electrical response value of the RI detector (μRIU). **(B)** (left) Standard curves and fitted linear regression equations for fructose, glucose, and sucrose (1–10 g/L). **(B)** (middle) The concentration of fructose, glucose, and sucrose in SSJ hydrolyzed by invertase from 0 to 24 h, calculated by substituting the peak area values in panel **(A)** (left) into the standard curve equation. **(B)** (right) The concentration of fructose, glucose, and sucrose in 1:1 dilution of SSJ hydrolyzed by invertase for 0–24 h as calculated by substituting the peak area values in panel **(A)** (right) into the standard curve equation. White columns represent fructose, gray diagonal columns denote glucose, and black columns indicate sucrose. **(C)** (left) Represents the particle size distribution of untreated SSJ, while **(C)** (right) The distribution after treatment. Gray columns represent the percentage of colloidal particles, blue columns represent the percentage of starch particles and macromolecules, and the red dotted line represents the cumulative distribution curve of suspended particle size.

In Figure 2A, the liquid chromatographic peaks depict the major sugar components in the hydrolysates of two SSJ feedstocks for 24 h enzymatic reactions. The peak areas for each sugar component in Figure 2A were substituted into the standard curve equations for fructose, glucose, and sucrose (Figure 2B, left), and the calculated values are plotted as bar graphs in Figure 2B (middle and right). In Figure 2A (left), the addition of invertase to the SSJ resulted in the hydrolysis of sucrose concentration from 59.59 to 1.02 g/L after 24 h. The ultimate reducing sugar content reached 133.69 g/L, comprising 63.09 g/L fructose and 70.60 g/L glucose. Since the total reducing sugar content of the raw sweet sorghum juice hydrolysate was quite high and could cause osmotic stress in clostridial cells, SSJ was diluted 1:1 before sucrose conversion (Song et al., 2010). Figure 2A (right) shows a decrease in sucrose levels from 31.93 to 0.55 g/L after the 1:1 dilution of SSJ following sucrose hydrolysis treatment, finally obtaining 33.59 g/L fructose and 37.73 g/L glucose. Based on the theoretical reaction equation for the hydrolysis of 1 M sucrose into 1 M fructose and 1 M glucose, the experimental data were substituted into Eq. (7) and Eq. (8):


(7) $$\begin{array}{l} \mathrm{Sucrose}\;\;\mathrm{hydrolysis}\;\;\mathrm{rate}=\\ \frac{initialsucroseconcentration\;-\;ultimatesucroseconcentration}{initialsucroseconcentration}\times 100\%\;\; \end{array}$$

(8) $$\begin{array}{l} \mathrm{Inversion rate of sucrose}=\\ \frac{ultimateinvertoseconcentration\;-\;initialinvertoseconcentration}{2\times \left(initialsucroseconcentration\;-\;ultimatesucroseconcentration\right)}\times 100\%\;\; \end{array}$$

The results indicated that the invertase-catalyzed sucrose hydrolysis rates of raw SSJ and 1:1 diluted SSJ surpassed 98%. The inversion rates of sucrose were 99.13 and 99.15%, and the sucrose loss rates were 0.87 and 0.85%, respectively. After 24 h, a trace amount of sucrose remained unhydrolyzed, which means there was no invertase residue at the end of the enzymatic reaction.

#### 3.1.2 First/second-order kinetic modeling of the sucrose enzymolysis reaction

**Figure 3:**
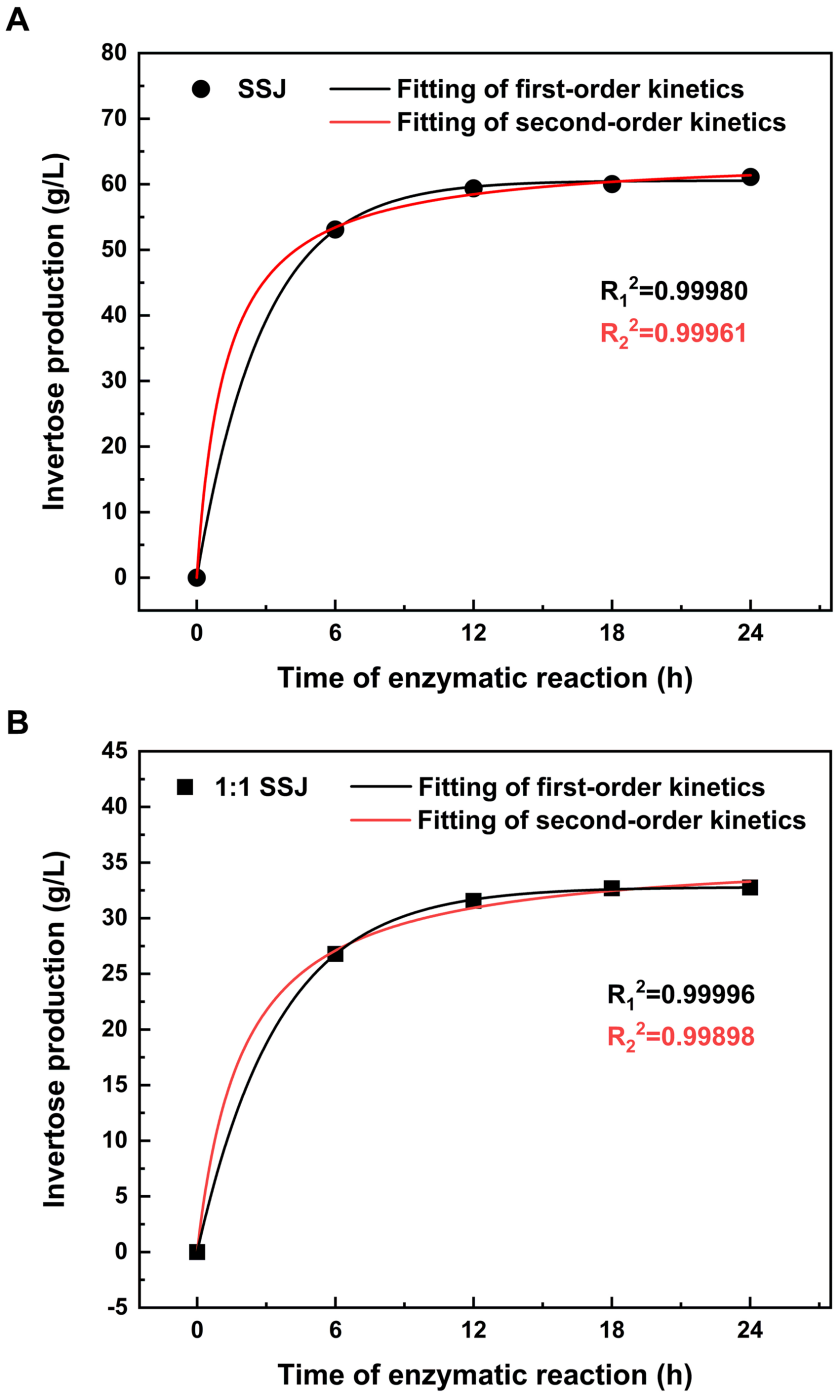
Kinetic modeling of non-reducing sugar hydrolysis in SSJ. Non-reducing sugars (sucrose) in SSJ were hydrolyzed at pH 5.0, temperature 42°C, and shaker speed 150 rpm. **(A)** The production of invertose over 0 to 24 h of sucrose hydrolysis in SSJ at an enzyme addition of 20 units/mL. **(B)** The production of invertose over 0 to 24 h of sucrose hydrolysis in a 1:1 dilution of SSJ at an enzyme addition of 10 units/mL. Circles and squares represent experimental values for raw SSJ and 1:1 diluted SSJ, respectively. The black solid line depicts the fitted curve of the first-order kinetic model, while the red solid line represents the fitted curve of the second-order kinetic model.

Both the first- and second-order kinetic models were fitted using the experimental data for sucrose conversion from SSJ in its original form or with a 1:1 dilution. The reaction rate was measured as the amount of invertose (i.e., fructose and glucose obtained by the inversion of sucrose) produced per unit time of sucrose hydrolysis (vertical axis in Figure 3). In Figure 3A, the first-and second-order kinetic fitting curves for the substrate hydrolyzed using SSJ are presented, accompanied by Eq. (9) and Eq. (10):


(9) $$y_{1}=60.14582-\exp\left(4.09677-0.35755\cdot x_{1}\right)\;\left(R_{1}{\;}^{2}=0.\mathrm{99980}\right)$$

(10) $$y_{2}=50.67681\cdot x_{2}/\left(1+0.78726\cdot x_{2}\right)\;\left(R_{2}{\;}^{2}=0\mathrm{.99961}\right)$$

Figure 3B shows the fitting curves of the primary and secondary kinetics using a 1:1 dilution of SSJ as the hydrolyzed substrate, with Eq. (11), Eq. (12) as follows:


(11) $$y_{3}=32.83666-\exp\left(3.49155-0.28181\cdot x_{3}\right)\left(R_{3}{\;}^{2}=0\mathrm{.99996}\right)$$

(12) $$y_{4}=17.14865\cdot x_{4}/\left(1+0.47234\cdot x_{4}\right)\;\left(R_{4}{\;}^{2}=0.\mathrm{99898}\right)$$

The final invertose concentration (*C_∞_*) released from sucrose hydrolysis of SSJ was predicted to be 60.15 and 64.37 g/L using the first−/second-order kinetic models, respectively. Additionally, the final invertose concentration of the 1:1 dilution of SSJ was predicted to be 32.84 and 36.31 g/L using the first-and second-order kinetic models, respectively. As shown in Figure 2B, the amount of invertose produced by the two SSJ substrates after 18 h of enzymatic reaction was not significantly different from that at 24 h. Therefore, a reaction time of 18 h can be considered the endpoint of the actual sucrose hydrolysis reaction. The ultimate invertose concentration was 60.04 g/L for SSJ and 32.69 g/L for the 1:1 dilution of SSJ, both slightly lower than the values predicted using the primary and secondary kinetic models.

#### 3.1.3 Particle size analysis in SSJ before/after treatment

The diameter range of colloidal particles is generally 0.001–1 μm (i.e., 1–1,000 nm) (Gregory, 2005), and a particle size range of 1–7.9 μm was used as a reference for starch particles (Alves et al., 2014). Taking its derivative concerning particle size, according to the cumulative distribution curve of suspended particles, the volume distribution of SSJ before and after treatment was determined as the percentage of particles in various particle size ranges, as shown in Figure 2C. The percentage change in colloidal particles before/after treatment is shown by the gray area in Figure 2C (left and right). Compared with the 18.57% percentage of colloidal particles (particle size <1 μm) in the untreated SSJ, the percentage of colloidal particles in the green-treated SSJ was reduced to 0.07%, and the colloidal particles removal rate reached 99.62%. The comparison of the volume percentage of particles with sizes range of 1–8.282 μm in two liquids showed that the green treatment process decreased the percentage of starch particles present in SSJ from 70.67 to 12.63%. Major suspended particles in SSJ varied from 0.1 to 10 μm, and the mean particle size was 2.73 μm. In green-treated SSJ, suspended particles were primarily in the range of 10–45 μm, representing 93.59% of particles<45 μm, and the median particle size increased to 18.53 μm compared to that of untreated SSJ.

#### 3.1.4 Changes in the concentration of harmful substances in SSJ before/after treatment

Apart from the reduction in polyphenol content, including substances such as benzaldehyde and 4-hydroxy-3-methoxy- (i.e., vanillin) after green treatment, there were noteworthy changes in the concentration of harmful substances. In total, 341 compounds were detected in SSJ both before and after treatment using ultra-performance liquid chromatography-mass spectrometry (UPLC-MS/MS), consisting of 191 positive ion modes and 150 negative ion modes. As shown in Supplementary Table S1, 1,2,3-Propanetricarboxylic acid, 2-hydroxy- (i.e., citric acid), l-phenylalanine, citrinin, 3-pyridinol, 2-methyl-and imidazol-1-yl-acetic acid were not detected in the treated SSJ. The percentages of dicarbonic acid, diethyl ester, and organic carbonate decreased from 1.254 to 0.883%, while the percentage of 1-propene-1,2,3-tricarboxylic acid, (E)- (i.e., trans-aconitic acid) decreased from 0.357 to 0.161%. The relative percentages of 2-propenoic acid, 3-(2-hydroxyphenyl)-, (E)- (i.e., o-coumaric acid) decreased by 0.397%, compared with those of untreated SSJ. The removal rates of benzene and its substituted derivatives, benzene, ethynyl- (i.e., phenylacetylene), benzaldehyde, and N-benzylformamide, reached 64.94, 66.00 and 37.84%, respectively. The removal of 2H-1,4-benzodiazepin-2-one, 7-chloro-1,3-dihydro-3-hydroxy-1-methyl-5-phenyl- (i.e., temazepam), 1H-purin-6-amine, and 1H-indole were 75.00, 84.63, and 48.89%, respectively.

### 3.2 Determination of optimal screening dose and screening of high-yielding butyric acid mutant strains after heavy-ion irradiation treatment

#### 3.2.1 MCMC modeling

**Figure 4:**
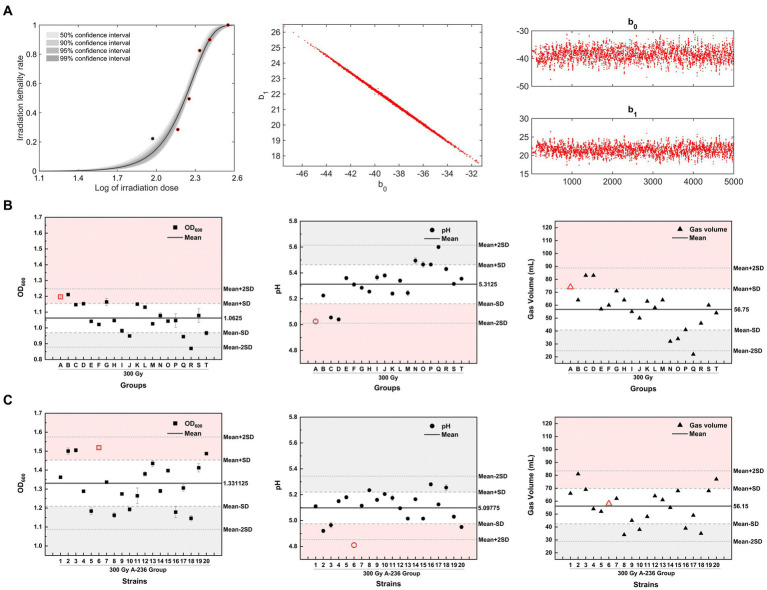
Heavy-ion irradiation combined with adaptive laboratory evolution screening for high-yielding butyric acid mutant strains. **(A)** (left) The MCMC model fit curve for *C. tyrobutyricum* Cirm BIA 2237 across varying heavy-ion irradiation doses. **(A)** (middle) A one-dimensional stationary distributed Markov chain, with a total chain length of 5 × 10^3^, and **(A)** (right) represents a two-dimensional Markov Monte Carlo chain, with 5,000 iterations. **(B,C)** The OD_600_, pH, and gas production of mutant strains of 300 Gy group A–T strains and generation IV strains, respectively, after liquid culture for 24 h (screening process is detailed in Supplementary Figure S1). The horizontal black thick line in the figure represents the mean value of the *Y*-axis parameter, the long gray dashed line represents the mean ± SD, while the short gray dashed line represents the mean ± 2 times the SD. Red areas represent positive mutation regions for the *Y*-axis parameter, and gray areas represent negative mutation regions for the *Y*-axis parameter.

Based on the previous finding that inducing microbial lethality in the 80–90% range is more likely to generate positive mutants with specific traits (Matuo et al., 2006), we constructed an MCMC model using log values of the irradiation dose and the corresponding lethality of *C. tyrobutyricum* Crim BIA 2237 as sample data. This model was used to evaluate the reliability of the sampled data for each dose by analyzing the 50, 90, 95, and 99% confidence intervals for each dose in a Bayesian framework. As shown in Figure 4A (left), the lethality rate of the strains increased with increasing doses of ^12^C^6+^ ion beam irradiation. The half-lethal dose (LD_50_) was approximately 180 Gy, whereas the lethality rate of the strains at a dose of 300 Gy was 85.3% and reached 100% after treatment with 360 Gy. The lethality rate corresponding to the log value (2.4771) of a 300 Gy dose fell precisely within the 99% confidence interval. Combined with the lethality rate of 85.3%, 300 Gy was selected as the optimal dose for screening. Figure 4A (middle) displays a one-dimensional Markov chain, whereas Figure 4A (right) displays a two-dimensional Markov chain of the model after 5,000 iterations. The stability, accuracy, and reliability of the model can be inferred from the one-dimensional parameter chain reaching a stationary distribution with a positive recurrent state.

#### 3.2.2 Screening for high-yielding butyric acid mutant strains

The strains screened after an irradiation dose of 300 Gy were divided into 20 groups and labeled alphabetically (A–T). For the directed screening of mutant strains, three parameters (OD_600_, pH, and gas production) of strains in liquid culture for 24 h from groups A–T were evaluated. The 300 Gy-A strain group, displaying the lowest pH (red circle in Figure 4B), was selected for further follow-up and screening, considering the weighting of the three screening parameters in descending order of pH > OD_600_ > gas production. Subsequently, 20 single colonies were picked from 300 Gy-A plates, numbered (1–20) in numerical order, and transferred to the treated SSJ medium for liquid culture. The strain with the lowest pH value in the liquid culture after 24 h was used for screening, which showed that the 300 Gy-A2 strain was a mutant strain of generation II. The mutant strains of generations I–IV were continuously screened using the aforementioned method. The resulting OD_600_, pH, and gas production after 24 h of liquid culture for these mutant strains are depicted as red icons in Supplementary Figures S1A–E. OD_600_ increased from 1.4225 for the 300 Gy-A2 strain in generation I to 1.5185 for the 300 Gy-A236 strain in generation IV; the pH value eventually decreased from 4.99 (generation I) to 4.81 (generation IV); gas production decreased continuously from 86 mL in Generation I to 58 mL in Generation IV. A mutant strain with high acid-producing capacity, high growth rate, and moderate gas production was obtained and named *C. tyrobutyricum* TGL-A236 (GenBank Accession Number: SUB14363947 Clostridium PP594088).

The Shapiro–Wilk test was conducted on three parameters: OD_600_, pH, and gas production of all strains from generations I to IV, supporting the original hypothesis that all data for the three parameters followed a normal distribution (*p* > 0.05). Approximately 68.7% of the observed values in the normal distribution model were within one standard deviation (SD) of the mean, whereas approximately 95.4% of the observed values were distributed within two SDs of the mean (Lee et al., 2015). In this study, above mean + SD (> 84.35% of data) and below mean-SD (< 34.35% of data) were used as criteria for identifying the group/strain with positive/negative mutations in that parameter (de Souza et al., 2023). The pH of the generation IV mutant strain *C. tyrobutyricum* TGL-A236 (red icons in Figure 4C) fell within the mean-2SD region. This result indicates that this mutant strain has a higher acid-producing capacity than that of the other 97.7% same-generation strains, providing a significant phenotypic advantage.

### 3.3 Effect of untreated/treated SSJ on *Clostridium tyrobutyricum* cells analyzed using the cytofluorescence double-staining assay

**Figure 5:**
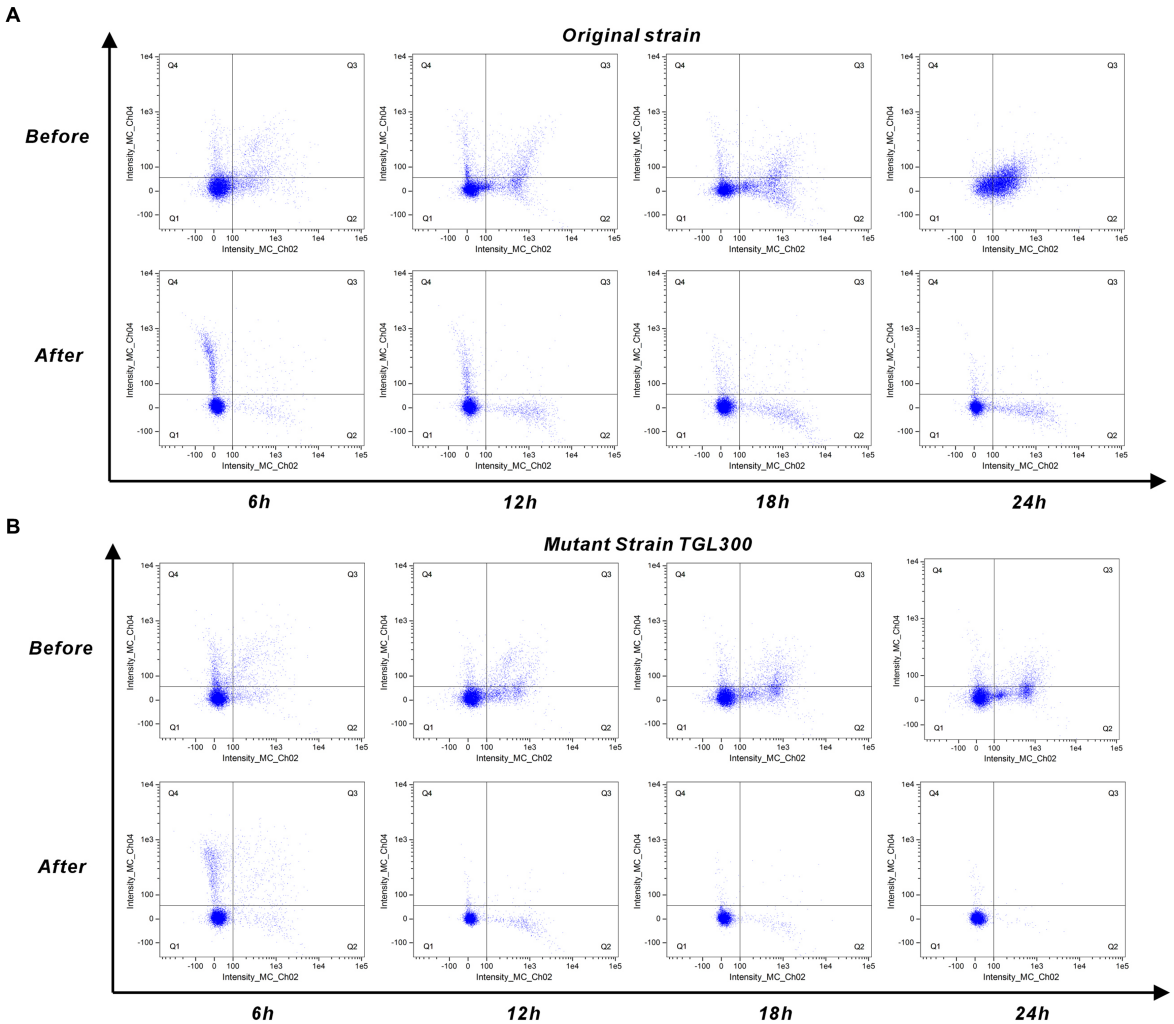
Cell staining of *C. tyrobutyricum* in pre/post-treated SSJ for 24 h. **(A)** Annexin V/PI double-staining assay results for the original strain *C. tyrobutyricum* Cirm BIA 2237 (5% v/v inoculum) in pre/post-treated SSJ. **(B)** Results of the mutant strain *C. tyrobutyricum* TGL-A236 under the same conditions. Results for the domesticated strain are shown in Supplementary Figure S2. Each sample involved the analysis of 10,050 ± 50 cells. Fluorescence channels Intensity_MC_Ch02 and Intensity_MC_Ch04 represent Annexin V-FITC and PI dye, respectively. Scatters in Q1 indicate normal cells, Q2 represents early apoptotic cells, Q3 signifies late apoptotic cells, and Q4 corresponds to necrotic cells.

Due to the too few cell quantities for 0 h, Figure 5 only shows the cellular response of the original strain and mutant strain to untreated/treated SSJ for 6–24 h. In Figure 5A, the percentage of early apoptotic cells (Q2 region) increased from 9.94 to 47%, and the percentage of late apoptotic cells (Q3 region) increased from 8.4 to 19.7% during the period of 6–24 h of inoculation of the original strain in untreated SSJ. In contrast, the percentage of early apoptotic cells increased from 2.86 to 9.28% in SSJ after treatment, while late apoptotic cells decreased to 0.36%. As shown in Figure 5B, the percentage of Q2 cells increased from 4.6 to 22.4%, and the percentage of Q3 cells increased from 5.48 to 7.73% after inoculation of mutant strains in untreated SSJ for 6–24 h. After being inoculation with SSJ treated for 24 h, the proportion of Q2 and Q3 cells in the mutant strain decreased to 0.39 and 0.04%, respectively. Normal cells (Q1 region) accounted for >99% of the samples. The rate of apoptosis for the domesticated strain in untreated/treated SSJ is illustrated in Supplementary Figure S2. The untreated SSJ significantly inhibited *C. tyrobutyricum* cell proliferation and induced apoptosis within 24 h. The rate of apoptosis increased over time and was mainly dominated by early apoptosis (Q2 region) and less dominated by late apoptosis (Q3 region). All three strains grew well in treated SSJ for 24 h, and the mutant strain had the highest proportion of normal cells.

### 3.4 Comparison of morphological changes of original/mutant strain cells in SSJ before/after treatment, and fermentation validation of the mutant strain

**Figure 6:**
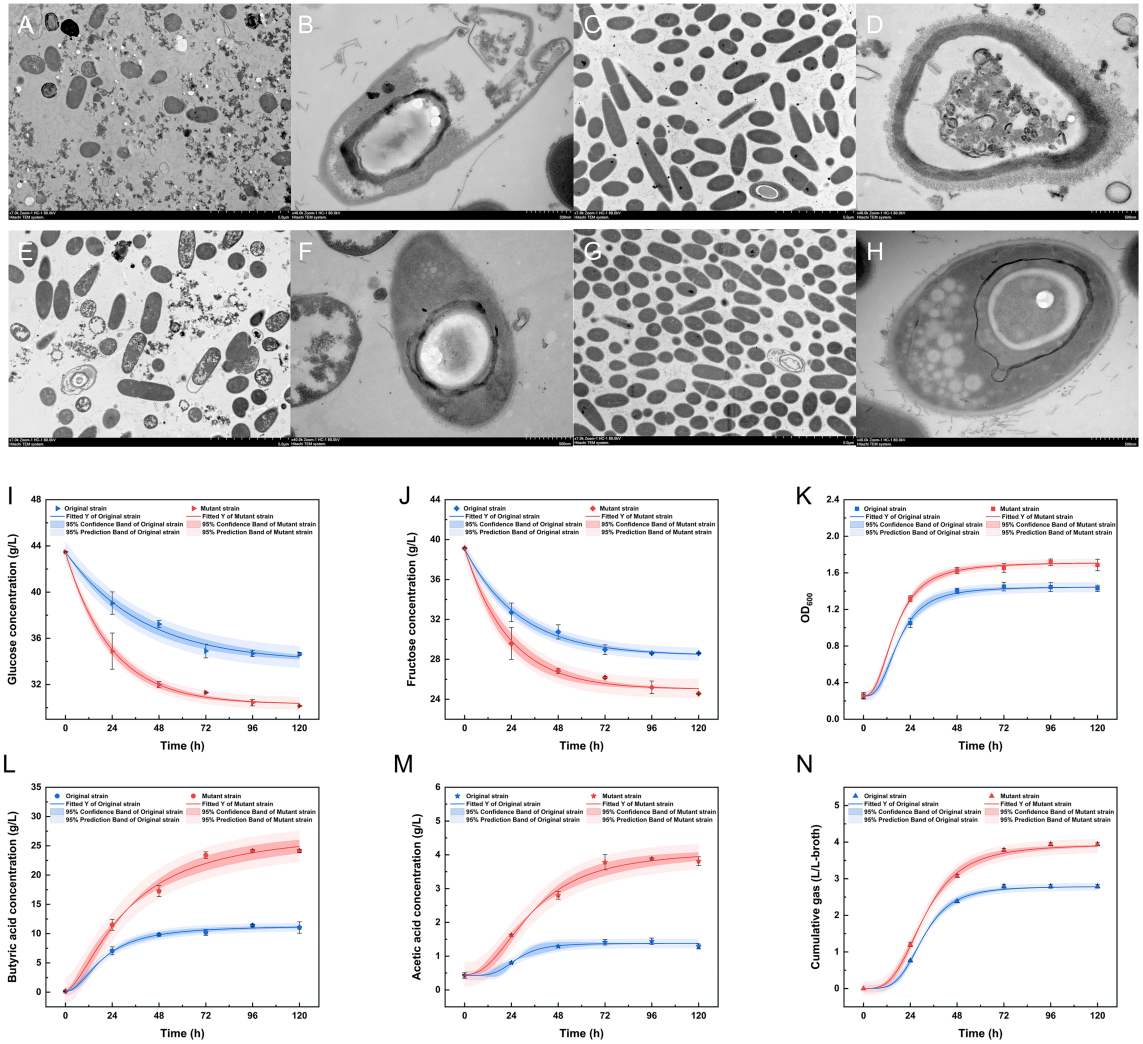
Cell morphology of the original strain and the mutant strain in untreated/treated SSJ and fermentation parameters in fermentation medium for 5 d. **(A–D)** Transmission electron microscopy (TEM) images of the original strain inoculated into untreated **(A,B)** and treated **(C,D)** SSJ medium for 18 h. **(E–H)** TEM images of the mutant strains inoculated into untreated **(E,F)** and treated **(G,H)** SSJ medium for 18 h. **(A,C,E,G)** Represent cell populations with a magnification of 7,000 ×, while **(B,D,F,H)** display individual cells with a magnification of 40,000 ×; the accelerating voltages were 80.0 kv. **(I–N)** The parameters of the fermentation of the original strain and the mutant strain in treated SSJ for 5 d; **(I)** shows the concentration of glucose (g/L); **(J)** shows the concentration of fructose (g/L); **(K)** shows the cell density (OD_600_); **(L)** shows the concentration of butyric acid (g/L); **(M)** shows the concentration of acetic acid (g/L); **(N)** shows the cumulative gas production (L/L-broth). The fitted curves in blue (original strain) and red (mutant strain) were obtained after nonlinear fitting based on the results of the sampling values. The dark blue and dark red regions signify the 95% confidence intervals of the fitted regression curves, and the light blue and light red regions represent the 95% prediction intervals of the fitted regression curves.

Biofermentation experiments were conducted using a 1:1 dilution of treated SSJ medium, and the 18-h time point was selected for transmission electron microscopy (TEM) to analyze the morphological changes of different strains in untreated/treated SSJ. In treated SSJ, both strains exhibited rod-like nutrient cell states, with the mutant strains (Figures 6C,G) displaying higher cell densities compared to the original strains (Figures 6A,E) at the same magnification. In untreated SSJ medium, the original strain cells (Figure 6B) lysed, showing disruption of cellular structure, while mutant strain cells (Figure 6F) maintained a relatively intact structure. In treated SSJ, original strain cells (Figure 6D) displayed intact structures, but with cell wall crumpling and invagination. Mutant strain cells during the acid-production phase (Figure 6H) exhibited a distinctive spindle-shaped morphology with a central bulge. Furthermore, developing endospore morphology, including ultrastructures such as bud wall, bud shell, cortex, nuclear core, and encapsulating white granules, was observed at the end of mutant strain cells.

Comparing the OD_600_ values (Figure 6K) of the original and mutant strains, the mutant strain reached a significantly higher maximum cell density (OD_600_ = 1.7163) compared with the original strain (OD_600_ = 1.4493). The changes in cumulative gas production (Figure 6N) were consistent with the growth curve. By 120 h, the original and mutant strains produced 2.7907 L and 3.9417 L of the CO_2_ and H_2_ mixture, respectively. The fructose and glucose concentrations of the mutant strain declined by 14.5630 and 13.3272 g/L, respectively, by the end of fermentation, 1.39 and 1.51 times higher than the fructose and glucose consumption rates in the fermentation broth of the original strain (Figures 6I,J). Throughout the fermentation process, the maximum butyric acid concentration of the mutant strain was 24.1364 g/L, which was 2.12 times higher than that of the wild-type strain. The acetic acid concentration of both strains reached its highest level at 96 h (1.4413 g/L for the original strain and 3.8798 g/L for the mutant strain) (Figures 6L,M).

### 3.5 Whole-genome sequencing and mutant gene analysis of *Clostridium tyrobutyricum* TGL-A236

**Figure 7:**
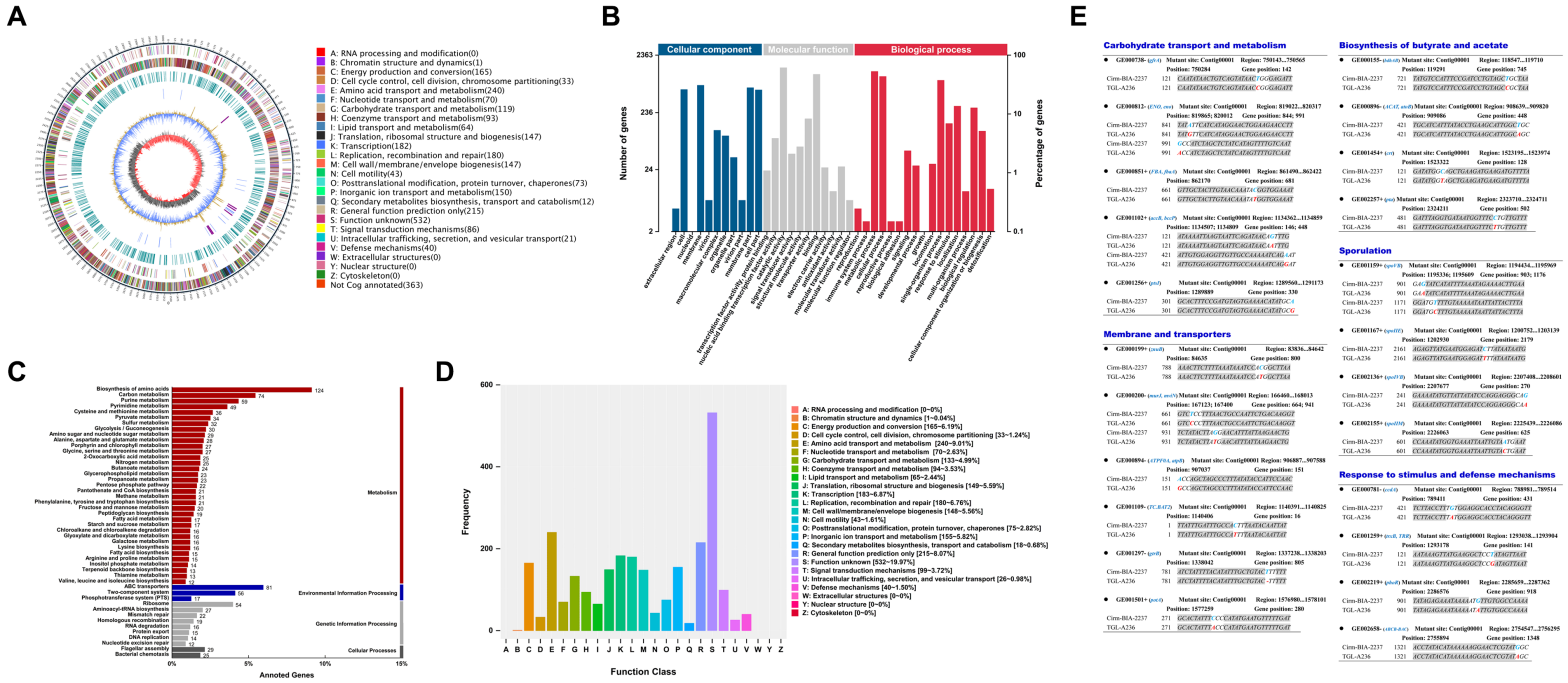
Genome atlas view, functional annotation, and mutated genes of the mutant strain *C. tyrobutyricum* TGL-A236 compared to the reference genome. **(A)** A genome-wide circle map of *C. tyrobutyricum* TGL-A236. **(B–D)** GO, KEGG, and eggNOG annotation classification maps, respectively, for variant genes in the mutant strain. **(E)** The mutant sites and base changes of mutant strain *C. tyrobutyricum* TGL-A236 compared with those of *C. tyrobutyricum* Cirm BIA 2237. Variant bases are highlighted in bold letters, blue bases are replaced by red bases, and “–” indicates a base deletion.

The genome-wide analysis of the mutant *C. tyrobutyricum* TGL-A236 (Figure 7A) reveals a circular chromosome of 3.02-Mbp with a GC content of 31.01%, basically aligning with previous reports (Munier et al., 2019). In Figure 7B, 2,363 genes in the *C. tyrobutyricum* TGL-A236 genome were annotated by the GO function, which were enriched in membranes and cells, catalytic activity, binding, and transporter activity, metabolic, cellular, and single-organism processes. As shown in Figure 7C, 1,360 genes were assigned KEGG Orthology numbers. The highest numbers of genes enriched in amino acid synthesis, ABC transporter proteins, carbon source metabolism, purine metabolism, and two-component systems were 124, 81, 74, 59, and 56, respectively. The functional annotation of eggNOG is shown in Figure 7D. Besides the R (general function prediction) proteins, the highest number of related proteins was in the following order: E (amino acid transport and metabolism; 240), K (transcription; 183), L (replication, recombination, and repair; 180), C (energy production and conversion; 165), and P (inorganic ion transport and metabolism; 155).

The mutational details for the mutant strain *C. tyrobutyricum* TGL-A236, as shown in Figure 7E. A total of 23 mutant genes were identified, covering carbohydrate transport and metabolism, membranes and transporters, biosynthesis of butyric acid and acetic acid, sporulation, and response to stimuli and defense mechanisms. Apart from synonymous mutations and one shift mutation due to a single-base deletion, noteworthy mutations include 10 base substitution mutations in non-synonymous coding regions, involving seven transitions (Ti) and three transversions (Tv).

### 3.6 Mutant gene expression levels detected using quantitative real-time polymerase chain reaction (qRT-PCR)

qRT-PCR was used to detect the relative expression levels of the mutant genes of *C. tyrobutyricum* TGL-A236. As shown in Figure 1B, all mutant genes were significantly upregulated except for *GE001297*, which was downregulated by the frameshift mutation. The gene *GE001102*, which is probably associated with carbohydrate transport and metabolism, had a relative expression 1.31 times higher than that of the control group (no significant difference). The relative expression of mutant genes like *GE000119*, *GE000200*, and *GE001501*, which are possibly involved in membrane and transport functions, was 8.95, 8.31, and 4.37 times higher than that of the original strain. The relative expression ratio of *GE001454*, a gene associated with the butyric acid synthesis pathway, was 2.66-fold higher than that of the control group. The transcription levels of gene *GE001167* (1.16-fold) and gene *GE002155* (1.62-fold), which probably encode phase II spore-forming protein E and protein M, were significantly upregulated. Meanwhile, the expression of genes *GE000781* and *GE002219*, which likely encode a hypothetical protein and a phosphate regulon sensor histidine kinase, respectively, was 1.30-fold and 4.76-fold higher than that of the control group. Amplification curves and melt curves from qRT-PCR experiments for all mutant genes are shown in Supplementary Figures S3–S13.

### 3.7 Pathway and process enrichment analysis of highly expressed mutant genes

**Figure 8:**
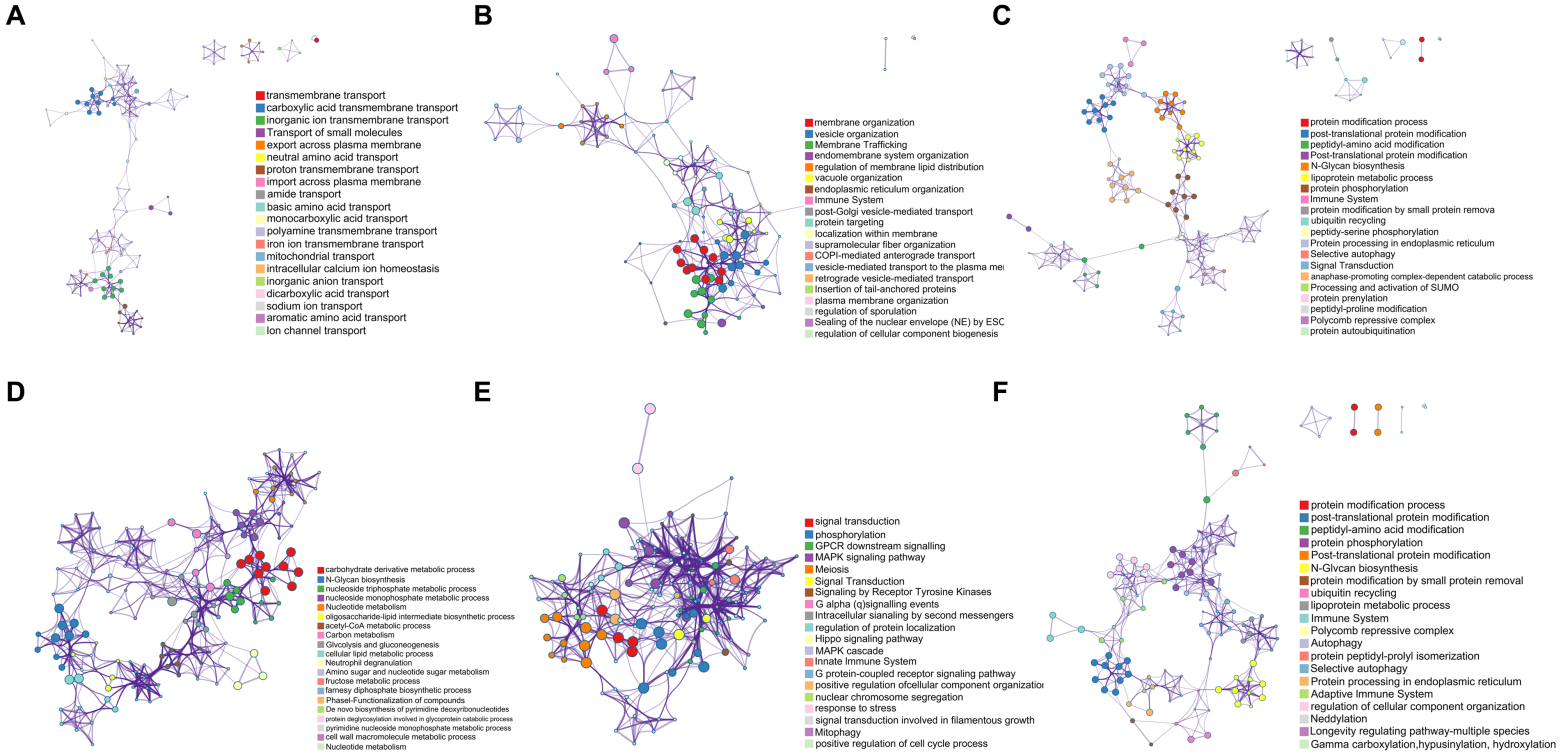
Pathway and process enrichment analysis results for each collected gene list in KEGG Pathway, GO Biological Processes, Reactome Gene Sets, Canonical Pathways, CORUM, WikiPathways, and PANTHER Pathway. All genes in the genome were used as the enrichment background. Terms with a *p*-value <0.01, a minimum count of 3, and an enrichment factor > 1.5 (the enrichment factor is defined as the ratio between the observed counts and the counts expected by chance) were collected and grouped into clusters based on their membership similarities. The most statistically significant term within a cluster was selected to represent the cluster. **(A)** The enrichment result of *GE000119*. **(B–D)** The enrichment results of *GE000200* under three different enrichment backgrounds, respectively. **(E,F)** The enrichment results of *GE002219* under two different enrichment backgrounds, respectively.

As shown in Figure 8A, the mutant gene *GE000119* had the highest GO slim frequency (65.71%) with GO:0055085 among the ontology sources, capturing a very high degree of similarity with GO:0051179 in terms of Biological Process (BP) and Molecular Function (MF) with GO:0051179, GO:0042592, and GO:0055085. Gene *GE000200* shared the same GO slim frequency of 66.74% in ontology sources under the three gene-enriched backgrounds GO:0071554, GO:0048856, and GO:1901135, and as shown in Figure 8B, captured in BP and MF categories with GO:0009987, GO:0051179, and GO:0061024 with the highest similarity. In Figure 8C, the highest similarity with GO:0008152, GO:0042592, and GO:0036211 was captured in terms of BP and MF, while in Figure 8D, the highest similarity to GO:0008152 and GO:1901135 was captured in BP and MF. Notably, *GE001454* had the highest GO slim frequency of GO:0006629 in ontology sources yet only 11.02% (data not listed). Surprisingly, gene *GE001501* has a GO slim frequency of 59.02% with GO:0055085 in ontology sources, similar to gene *GE000119* (data not listed). Finally, the highest GO slim frequency of gene *GE002219* in ontology sources under two gene-enriched backgrounds GO:0023052 and GO:0036211 were 61.36 and 59.14%, respectively. As shown in Figure 8E, a very high similarity to GO:0050896, GO:0050789, and GO:0007165 was captured in the BP aspect with the MF aspect. Figure 8F shows a high similarity with GO:0008152, GO:0042592, and GO:0036211 in terms of BP and MF.

### 3.8 Homology modeling and MolProbity results analysis of proteins encoded by highly expressed mutant genes

**Figure 9:**
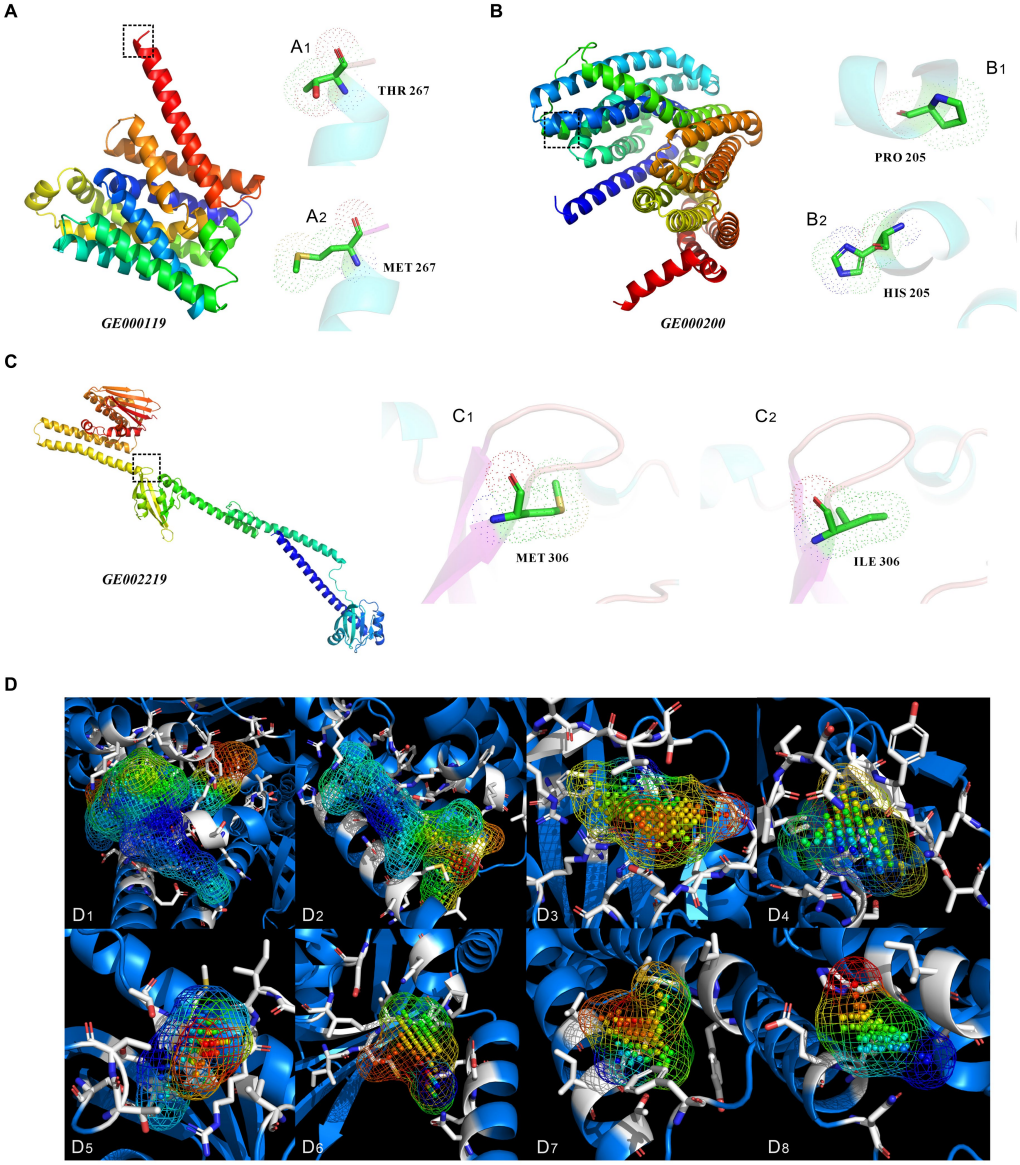
Three-dimensional structure prediction results analysis of proteins encoded by non-synonymous mutant genes, and protein cavities of mutated genes *GE002219*. **(A–C)** Three-dimensional structures of proteins encoded by the mutant genes of *GE000119*, *GE000200*, and *GE002219*, respectively. **(A**_1_**,A**_2_**,B**_1_**,B**_2_**,C**_1_**,C**_2_**)** show the specific locations and types of amino acid site changes between the primitive and mutant genes of *GE000119*, *GE000200*, and *GE002219*, respectively. **(D)** The protein cavities of mutated genes *GE002219* and their coulomb potential and hydrophobicity (only showing cavities with a volume greater than 100 Å^3). The following are the volume of the cavity and the point (atom) number of each cavity. **(D**_1_**)** 1195 Å^3, 840 pts. **(D**_2_**)** 715 Å^3, 403 pts. **(D**_3_**)** 287 Å^3, 196 pts. **(D**_4_**)** 192 Å^3, 102 pts. **(D**_5_**)** 165 Å^3, 89 pts. **(D**_6_**)** 185 Å^3, 80 pts. **(D**_7_**)** 167 Å^3, 77 pts. **(D**_8_**)** 125 Å^3, 60 pts. The above MolProbity results, Ramachandran plot, and specific locations of the cavities are detailed in Supplementary Figures S14–S19.

Changes in encoded protein structure were predicted via protein homology modeling for the four highly expressed non-synonymous variant genes with GO slim frequency higher than 50% (Figure 9). Meanwhile, the MolProbity results were also analyzed offering quality validation for 3D structures of predicted proteins. The gene *GE000119* in Figure 9A has 100% sequence identity with W6N5G3.1.A (Zinc ABC transporter, inner membrane permease protein ZnuB), with a MolProbity score of 0.59, indicating that the two share the same domain. However, the protein sequence similarity between this gene of the original strain and W6N5G3.1.A was 99.63%. The number of bad bonds in the protein encoded by gene *GE000119* changed from 0/2073 to 0/2074 compared with the predicted protein structure of the original strain. The best match of the mutant gene *GE000200* protein sequence resulted in W6N5X4.1. A Probable lipid II flippase MurJ AlphaFold DB model of W6N5X4_CLOTY (Figure 9B), with a MolProbity Score of 0.76. The protein structure changed from 0/4133 to 0/4136 for bad bonds and from 18/5609 to 18/5612 for bad angles based on the original reference protein. The highest GO slim frequency of the *GE001454* variant gene was only 11.02% in the orthologous group, hence homologous modeling was not carried out. Unexpectedly, the protein sequences encoded by *GE001501*, and the protein sequence encoded by this gene of the original strain, were all similar to the reference protein (W6NBK4.1.A Spermidine/putrescine import ATP-binding protein PotA AlphaFold DB model of W6NBK4_ CLOTY) with 100% sequence identity. The non-synonymous mutation site of this gene did not alter the functional domain of the encoded protein; therefore, the homology modeling results of *GE001501* are not presented in this study. The protein sequence and structure predicted from the variant gene *GE002219* (Figure 9C) showed a Seq Identity of 99.82% with A0A4P7ZZ09.1.A histidine kinase AlphaFold DB model of A0A4P7ZZ09_CLOTY. The MolProbity Score was 0.58, and the Ramachandran Favored had a residual of 98.58%. A comparison with the original reference protein structure revealed that the bad angles changed from 16/6225 to 13/6226. Since the homologous protein A0A4P7ZZ09.1.A of *GE002219* was not available in the UniProtKB database, the protein cavities and amino acid residues of the modeled proteins were classified in this study based on their Coulomb potential and hydrophobicity. In Figure 9D, there are a total of 47 protein cavities (protein active pockets), the volume of the cavities is of the highest order of magnitude of 1,195 Å^3 and the lowest order of magnitude of 22 Å^3, and the highest distribution of the number of points (atoms) in the interior of the cavities is 840 pts. and the lowest distribution of the number of points (atoms) in the interior of the cavities is 4 pts. The simulation and calculation were conducted on the first eight protein cavities with volumes greater than 100 Å^3 (Figures 9D_1_–D_8_), and the locations of these cavities are detailed in Supplementary Figure S19.

## 4 Discussion

The sugar fraction content of untreated SSJ measured using HPLC (Figure 2) showed that SSJ and 1:1 diluted SSJ contained 59.59 g/L and 31.93 g/L sucrose, respectively. To convert the high percentage of sucrose in SSJ into fructose and glucose that can be directly utilized by *C. tyrobutyricum* and explore greener pretreatment methods for SSJ in food (sweeteners, syrups) production, this study employed an enzymatic method to convert sucrose and an enzyme kinetic modeling to evaluate the hydrolysis effect. According to the high fit in both primary-order (*R*^2^ > 0.999) and secondary-order (*R*^2^ > 0.99) kinetic model for sucrose enzymatic reactions of the two SSJ substrates, and the pH of hydrolysate was higher than the invertase’s isoelectric point (PI 3.4–4.4), the reasonability of enzyme amount was confirmed, preventing invertase crystallization (Colonna et al., 1975). The first-order kinetic model with a larger coefficient of determination (R^2^) was used to calculate the rate constant for reaction hydrolysis (*K_reaction_*) under consistent conditions for the enzymatic reactions of the two substrates: 0.35755 h^−1^ for SSJ and 0.28181 h^−1^ for 1:1 diluted SSJ. The rate constant reaction hydrolysis for this enzymatic reaction was greater than the *K_reaction_* results reported by de Souza Soares et al. (2019) for invertase hydrolysis of a 600 g/L sucrose solution at pH ~5.0 and 40°C (0.27 ± 0.02). It has been reported that enzymatic sucrose hydrolysis can also be combined with various techniques, such as ultrasonication and immobilization (Kotwal and Shankar, 2009), but the present process is more economical in ensuring sucrose hydrolysis rate (>98%) and suitable for pretreatment and large-scale industrial production of SSJ as a food ingredient, consistent with the green concept of “carbon neutrality.”

From the results of the laser particle size analyzer (Figure 2C), 99.62% of the colloids (<1 μm) and 82.13% of the starch particles (1–8.282 μm) were removed from the SSJ after adding diatomite. This is because diatomite can adsorb coagulated colloids, calcium phosphate flocs, and starch particles in the sterilized SSJ, thus forming stable large flocs centered on diatomite particles. PO43-and Ca_2+_ ions, naturally present in SSJ, are prerequisites for the formation of calcium phosphate-bridged flocs during juice heating. Positively charged calcium ions interact with the particles in SSJ, changing the zeta potential of the particles and thus destabilizing the colloids. Diatomite adsorbs and increases the weight of the colloidal particles and promotes particle aggregation to expedite sedimentation and recovery (Andrzejewski et al., 2013a). The removal effect of colloid and starch particles was also observed in the electron microscopic view. Flocculent and granular impurities dispersed in the background of untreated SSJ slices (Figures 6A,E), contrasting with treated slices (Figures 6C,G) where impurities were notably reduced. As the results of the pre−/post-treatment SSJ measurements using UPLC-MS/MS (Supplementary Table S1), the diatomite treatment effectively decreased the relative levels of several substances with high toxicity in the SSJ: e.g., dicarbonic acid, diethyl ester (1.254–0.883%), phenylacetylene (1.158–0.406%), 1H-indole (from 0.090–0.046%), benzaldehyde (0.050–0.017%), citrinin (0.034% to non-detect), temazepam (0.016–0.004%), and 1-Dodecanamine, N, N-dimethyl-, N-oxide (0.031–0.015%). Toxicological information such as median lethal dose (LD_50_), median effect dose (EC_50_), and chronic value (ChV) for these substances are available on the Environmental Protection Agency website.^4^ Larger LogK_ow_ values for these substances (Supplementary Table S1) signify a stronger potential for substances to reach cellular targets through the biofilm, generating baseline toxicity in the biofilm channel, which is accompanied by migration and cumulative effects during the growth of strains to exhibit acute/chronic toxicity (Qin et al., 2010). The physiological and biochemical effects of untreated SSJ on *C. tyrobutyricum* are shown in Figure 6F, where the cells of the original strain at 18 h showed a disruption of structural integrity, whereas the cells of the original strain in the treated SSJ were in good condition and had a greater cell density. Cell staining of the original strain in treated SSJ at the same time showed a 21.2% increase in the proportion of normal cells (86.9%) compared with that in untreated SSJ (65.7%) (Figure 5A). The above results verified the decolloid and detoxification effect of the process. Previous SSJ treatments commonly used milk of lime (MOL) in combination with polyanionic flocculants to reduce juice turbidity and impurities (Andrzejewski et al., 2013b), but these chemicals tend to introduce exogenous impurities that further complicate the treated SSJ fraction, hindering SSJ application in the food industry. Therefore, this study presents a cost-effective, safe, and feasible treatment process for the environmentally friendly production and treatment of SSJ in primary processing and biological products in the food industry.

The study focused on enhancing the integrative properties of *Clostridium tyrobutyricum* through a 300 Gy dose of ^12^C^6+^ ion beam irradiation mutagenesis method, based on the Markov Chain Monte Carlo model and Bayesian framework. As shown in Supplementary material, 20 random colonies in the plate of bacterial liquid after 300 Gy irradiation treatment were used as I generation mutant strains (Supplementary Figure S1A). The strain with the lowest pH value of the bacterial liquid after 24 h of liquid cultivation was selected as the next-generation mutant strain, and the mutant strain TGL-A236 was successfully selected through the adaptive evolution and directional screening by cultivating IV generation of the mutant strain in the “solid–liquid” culture of the SSJ medium (Supplementary Figures S1B–E). As shown in Supplementary Figures S1A–E, 20 random colonies in the plate of bacterial liquid after 300 Gy irradiation treatment were used as I generation mutant strains (Supplementary Figure S1A), and the strain with the lowest pH value of the bacteria suspension after 24 h of liquid cultivation was selected as the next generation mutant strain. The mutant strain TGL-A236 was successfully selected through the adaptive evolution and directional screening by cultivating IV generations of the mutant strain in the “solid–liquid” culture of SSJ medium (Supplementary Figures S1B–E). The growth and acid-producing properties of the mutant strain can be verified from the results of fermentation kinetics (Figures 6I–N): the maximum cell density of the mutant strain (OD_600_ = 1.7163) was significantly higher than that of the original strain (OD_600_ = 1.4493). Moreover, the maximum butyric acid concentration of the mutant strain (24.1364 g/L) was 2.12 times that of the original strain (11.4020 g/L) throughout the whole fermentation process using the treated SSJ as the carbon source. Therefore, the growth performance, acid production, and butyric acid tolerance of the mutant strain were significantly greater than those of the original strain. Additionally, the effects of heavy ion beam irradiation on the physiological and biochemical characteristics of mutant strains can be confirmed by observing the changes in cell morphology and apoptosis rate. The integrity of the cellular structure of most original strain cells was only observed in the treated SSJ (Figure 6D), whereas the mutant strain cells could maintain normal morphology and greater densities even in untreated SSJ for 18 h (Figure 6F); thus, the mutant strains were more resistant to colloid and toxic substances in the untreated SSJ. The percentage of normal cells of the mutant strain in the treated SSJ after 18 h was as high as 97.3%, significantly higher than that of normal cells of the original strain in the untreated and treated SSJ (86.9 and 65.7%, respectively), consistent with the observation of TEM and the trend of the growth curve of the fermentation. The production of butyric acid by *Clostridium tyrobutyricum* using sweet sorghum juice as the sole substrate without the addition of any exogenous substances has not been previously reported. Therefore, the heavy ion beam irradiation mutagenesis breeding strategy of *C. tyrobutyricum* mutants proposed in this study provides strong data support for breeding food microorganisms in *Clostridium* spp.

For a deeper understanding of *C. tyrobutyricum* TGL-A236, 23 mutation sites were screened in mutant TGL-A236 (Figure 7E). This study focused on non-synonymous mutant genes that can change amino acid sequences on the polypeptide chain. A total of 10 non-synonymous mutant genes were identified by analyzing the codon changes of encoded amino acids in these CDS sequences, namely, *GE000119*, *GE000200*, *GE000781*, *GE001102*, *GE001167*, *GE001297*, *GE001454*, *GE001501*, *GE002155*, and *GE002219*. Based on the results of qRT-PCR on the relative expression of the above 10 non-synonymous mutant genes in mutant TGL-A236 (Figure 1B), we focused on the top 5 mutant genes with the highest expression fold changes, namely *GE000119* (8.95-fold), *GE000200* (8.31-fold), *GE002219* (4.76-fold), *GE001501* (4.37-fold), and *GE001454* (2.66-fold). Combining the results of pathway and process enrichment analysis (Figure 8) and protein homology modeling (Figure 9) can reveal the biological processes, specific functions, and structural changes of the encoded proteins of these five mutated genes. The protein structure predicted by mutant gene *GE000119* was most similar to W6N5G3.1.A, which was zinc transport system permease protein ZnuB involved in the ABC transporter protein pathway (ko02010). This protein is highly associated with the homeostatic process (GO:0042592) and transmembrane transport (GO:0055085) functions in the top-level Gene Ontology. The high expression of gene ZnuB in the mutant strain maintained zinc homeostasis in the periplasm and normal cell growth and thus exhibited enhanced growth performance over the original strain in fermentation experiments (Peng et al., 2021). The gene *GE000200* is probably responsible for encoding the putative peptidoglycan lipid II flippase protein (W6N5X4.1.A), shown in Figure 8 to be involved in cellular process (GO:0009987) and membrane organization (GO:0061024) functions, is associated with the peptidoglycan synthesis pathway (ko00550) in KEGG. The encoded enzyme of this gene may be responsible for the translocation of lipid-linked peptidoglycan precursors to the nascent site of cell wall synthesis, a key gene for cell wall biosynthesis (Huber et al., 2009). Thus, TEM observation that mutant strains in both untreated or treated SSJ exhibited enhanced cell wall integrity is likely to be related to the significant upregulation of the expression of gene *GE000200*. Gene *GE001501* was modeled and aligned for protein homology and had the highest similarity to W6NBK4.1.A (Figure 9D). It is the sarcosine/putrescine transporter protein complex potA in the ABC transporter (ko02010) pathway and associated with E class “Amino acid transport and metabolism” in the COG (cluster of orthologous groups of proteins) database. PotA facilitates spermine entry into the cytoplasm via transmembrane transport by binding to ATP to provide energy coupling, mediates the stress response of mutant strain cells to environmental stress, and enhances stress resistance to toxic stress in SSJ (Emerson et al., 2008). Thus, the mutant strain could maintain 76.7% of normal cell proportion even after 12 h in untreated SSJ.

The mutant gene *GE001454* may be involved in the butanoate metabolism (ko00650) and carbon metabolism (ko01200) pathways in KEGG, and is classified as a class I “Lipid transport and metabolism” function in COG. The predicted protein sequence is most similar to 5z7r.1.D, a short-chain enoyl CoA hydratase (EC:4.2.1.17). This enzyme assists in the intracellular carbon flux of *C. tyrobutyricum* toward butyric acid synthesis. Therefore, the significant upregulation of this gene is consistent with the experimental result that the final butyric acid concentration and carbon source utilization of the mutant strain TGL-A236 are more than 1-fold higher than that of the original strain. The predicted protein structure of gene *GE002219* in Figure 9C shows the highest similarity to histidine kinase phoR (A0A4P7ZZ09.1.A), a sensor of the phosphate-dependent two-component regulatory system. This gene function was categorized in the Gene Ontology analysis as a response to stimulus (GO:0050896) and regulation of biological process (GO:0050789) and annotated in COG and KEGG to class T “Signal transduction mechanisms” and two-component (ko02020) pathway, respectively. Most of the PO_4_^3−^ ions in SSJ can be removed by sterilization and diatomite treatment in this study. Mutant strain cells might employ the sensor kinase phoR to detect low extracellular phosphate concentrations through the N-terminal sensor structural domain positioned between the two transmembrane structural domains. This initiates a conformational change that activates the C-terminal autokinase structural domain, leading to autophosphorylation of specific conserved histidine residues. The phosphate moiety is then transferred to the intracellular response regulator phoP, facilitating extracellular phosphate uptake. Besides, the greater robustness of *C. tyrobutyricum* TGL-A236 during endospore formation and granule synthesis in scanning electron microscopy may be linked to structural alterations induced by variations in the phoR gene (Fiedler et al., 2008).

To analyze the “3D structure–function” relationships of target proteins encoded by non-synonymous mutant genes, residues in the protein sequences encoded by highly expressed mutant genes were mapped to residues in homologous template proteins for alignment and homology modeling. The change in protein structure of the mutant gene *GE000119* is shown in Figure 9A. The active site in the α-helical region highlighted in the box changes from the polar uncharged/polar neutral amino acid THR 267 (A_1_) to the nonpolar amino acid residue MET 267 (A_2_), which tends to encapsulate the side chains of this region inside the protein to form a hydrophobic core that stabilizes the protein structure (Kyte and Doolittle, 1982). This protein belongs to the ABC-3 integral membrane protein family involved in the intake and regulation of zinc in the periplasm of bacterial strains. It has been reported that high zinc concentration environments significantly increase the number of copies of antimicrobial resistance genes in bacteria. Therefore, the mutant strains are more resistant to SSJ toxicants and likely to be related to the high expression of gene *GE000119* (Peng et al., 2021). Figure 9B demonstrates a conformational change of the *GE000200* gene-encoded protein, where the nonpolar amino acid PRO 205 (B_1_) in the blue α-helix region changes to the positively charged amino acid HIS 205 (B_2_). This protein is a member of the MurJ/MviN family associated with peptidoglycan biosynthesis, thus upregulation of MurJ gene expression possibly allows mutant strain cells to maintain a certain mechanical resistance in SSJ containing various types of inhibitors, thereby maintaining the normal structure and shape of the cells in TEM view (Sham et al., 2014). When attempting to model the protein homology of *GE001454*, we found that multiple parameters of its MolProbity Results and the residue positions of several active sites were drastically changed compared with the original reference protein structure. It did not bring about changes in the conformational and functional structural domains of the mutant gene *GE001501* encoded protein, while significantly increasing the relative expression level of the encoded protein (Figure 1); however, the exact mechanism remains nebulous. More detailed analysis and characterization of the structures of the proteins encoded by these two mutant genes remain warranted, in combination with X-ray crystalline diffraction and nuclear magnetic resonance. Notably, data regarding reference protein A0A4P7ZZ09.1.A predicted by gene *GE002219* (such as the protein family and the source of the organism) is not available in the UniProtKB database, hence the specific function of its encoded protein remains ambiguous. As shown in Figure 9C, the change in the functional structural domain of the gene *GE002219* encoded protein is located in the β-folded region adjacent to the LOOP region of the box in the figure, where the active site residue changes from the nonpolar amino acid MET 306 (C_1_) to the nonpolar amino acid ILE 306 (C_2_). A model of the framework of the protein encoded by gene *GE002219* can be constructed and the protein cavities it forms can be predicted based on its Coulomb potential and hydrophobicity (Nachliel and Gutman, 2023). The Coulomb potential is shown as an irregular grid region in Figure 9D, where the points inside the grid are atomic/electronic arrangements and the rods surrounding the grid are modeled as the spatial conformation of the amino acid residues in the vicinity of this cavity. Among the 47 protein cavities identified, 8 with volumes larger than 100 Å^3 were screened (Figures 9D_1_–D_8_): (D_1_) is a cavity wrapped by 38 amino acid residues containing 840 pts. of atoms and forms a cavity volume of 1,195 Å^3. The total number of amino acid residues outside the cavity shown in (D_2_) is 23 with 403 pts. atoms, with a cavity volume of 715 Å^3. The cavity (D_3_) is surrounded by 15 amino acid residues with 196 pts. atoms and the cavity volume is 287 Å^3. The cavity (D_4_) has a volume of 192 Å^3 and is wrapped by 13 amino acid residues with 102 pts. atoms. The cavity (D_5_) is similarly wrapped by 13 residues and has a volume of 165 Å^3 and 89 pts. atoms. The cavity (D_6_) is enveloped by 12 amino acid residues with a total of 80 pts. of atoms and a volume of 185 Å^3. The wrapped cavity (D_7_) has 8 amino acid residues with a total of 77 pts. of atoms and a volume of 167 Å^3. (D_8_) has a minimum cavity volume of 125 Å^3 and is wrapped by 6 amino acid residues with a total of 60 pts. of atoms. These protein spaces display pocket shapes with small openings and large bellies, as well as irregular shapes such as tubular, grooved, and shallow pits, which are molecular structures capable of holding a certain volume. These docking pockets are located at the actual active binding site; there is a higher probability of finding the correct active conformation and binding mode of the ligand (Xue et al., 2019). Therefore, the protein cavities D_1_–D_8_ with a volume greater than 100 Å^3 are a crucial foundation and priority for the subsequent stage: searching for docking pockets/recognizing binding sites.

In summary, this study provided a comprehensive analysis and prediction of the protein structure encoded by the above highly expressed non-synonymous mutant genes *GE000119*, *GE000200*, and *GE002219*. However, other differentially-expressed genes encoded protein structures and functions need to be further investigated in our future work due to the large amount of workload. This study provides valuable data and directional support for understanding the nature and mode of binding of protein active sites to cofactor/metal ions and similarity analysis with the annotated protein folds that may be in the catalytic and regulatory sites, and promotes the in-depth development of *Clostridia* microorganisms for food industry applications in the future.

## 5 Conclusion

This study, for the first time, involved the management and utilization of a renewable biomass, sweet sorghum juice (food raw materials), for food additives (bio-butyric acid) production. This work integrated food raw materials, food microbiology, and food additives, addressed challenges in conventional methods, and presented an innovative and comprehensive process strategy to enhance sweet sorghum juice and *Clostridium tyrobutyricum* strain development for bio-butyric acid production.

## Data availability statement

The datasets presented in this study can be found in online repositories. The names of the repository/repositories and accession number(s) can be found in the article/Supplementary material.

## Author contributions

M-HL: Conceptualization, Data curation, Formal analysis, Methodology, Software, Writing – original draft. XZ: Conceptualization, Data curation, Formal analysis, Funding acquisition, Investigation, Project administration, Resources, Software, Supervision, Validation, Visualization, Writing – review & editing. M-MZ: Data curation, Formal analysis, Methodology, Software, Writing – original draft. Y-JW: Data curation, Formal analysis, Methodology, Software, Writing – original draft. BZ: Formal analysis, Funding acquisition, Investigation, Project administration, Resources, Supervision, Visualization, Writing – original draft. ND: Data curation, Formal analysis, Methodology, Software, Writing – original draft. Q-FW: Data curation, Formal analysis, Methodology, Software, Writing – original draft. C-RL: Data curation, Formal analysis, Methodology, Software, Writing – original draft. Z-YD: Data curation, Formal analysis, Methodology, Software, Writing – original draft. J-LR: Data curation, Formal analysis, Methodology, Software, Writing – original draft. J-RZ: Data curation, Formal analysis, Methodology, Software, Writing – original draft. C-LJ: Data curation, Formal analysis, Methodology, Software, Writing – original draft. JL: Data curation, Formal analysis, Methodology, Software, Supervision, Writing – original draft. DL: Funding acquisition, Project administration, Resources, Supervision, Validation, Visualization, Writing – review & editing. H-YZ: Funding acquisition, Project administration, Resources, Supervision, Validation, Visualization, Writing – review & editing.

## Footnotes

^1^ http://geneontology.org/

^2^ https://www.kegg.jp/

^3^ http://eggnogdb.embl.de/

^4^ https://www.epa.gov/
