# Supplementary material for: Integration of food raw materials, food microbiology, and food additives: systematic research and comprehensive insights into sweet sorghum juice, Clostridium tyrobutyricum TGL-A236 and bio-butyric acid
Source: Front Microbiol. 2024 May 30;15:1410968. doi: 10.3389/fmicb.2024.1410968 (PMC11169884; doi:10.3389/fmicb.2024.1410968)
Supplement: Supplementary file 1 [file Data_Sheet_1.PDF]

Supplementary Material

1 Supplementary Figures and Tables

1.1 Supplementary Figures

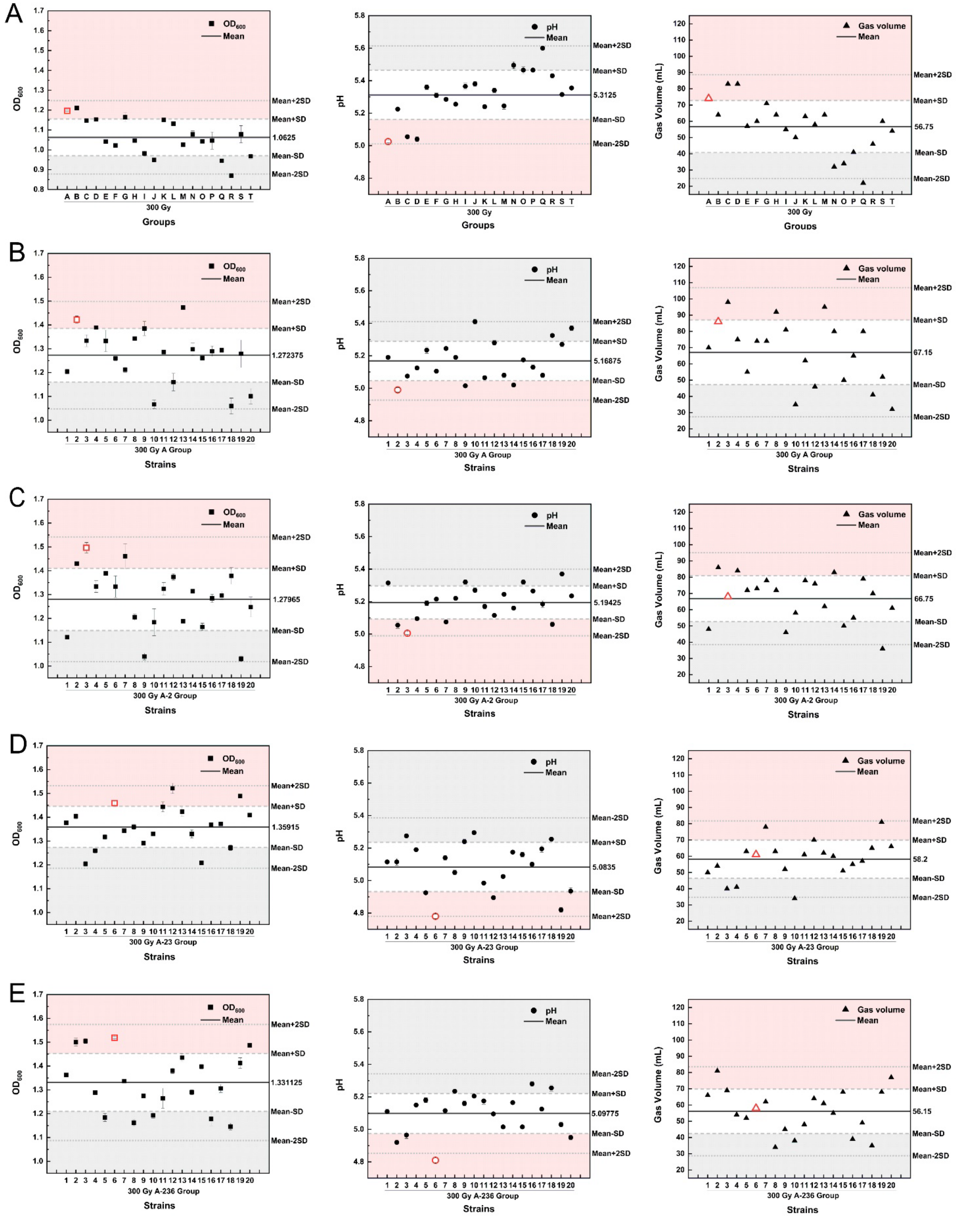

Supplementary Figure S1. Screening for high-yielding butyric acid mutant strains after liquid culture for 24 h following 300 Gy $^{12}C^{6+}$ ion irradiation treatment. A, B, C, D, and **E** represent the

OD_{600}, pH, and gas production of mutant strains of group A–T, generation I, generation II, generation III, and generation IV, respectively.

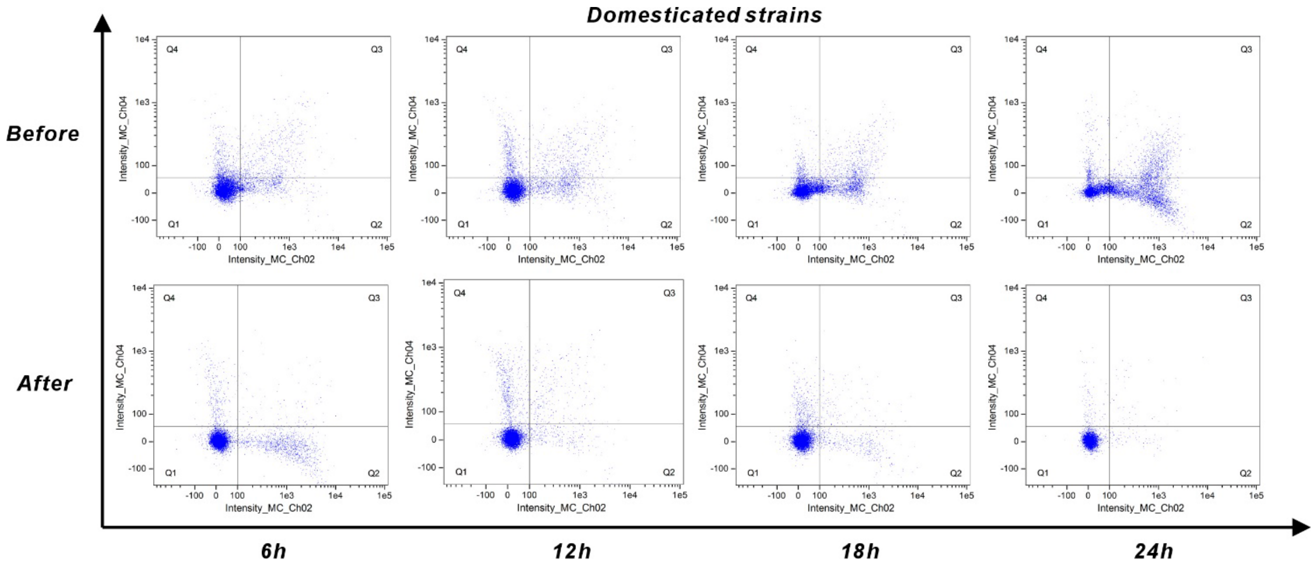

Supplementary Figure S2. Cell staining of *C. tyrobutyricum* domesticated strain in pre/post-treated SSJ for 24 h.

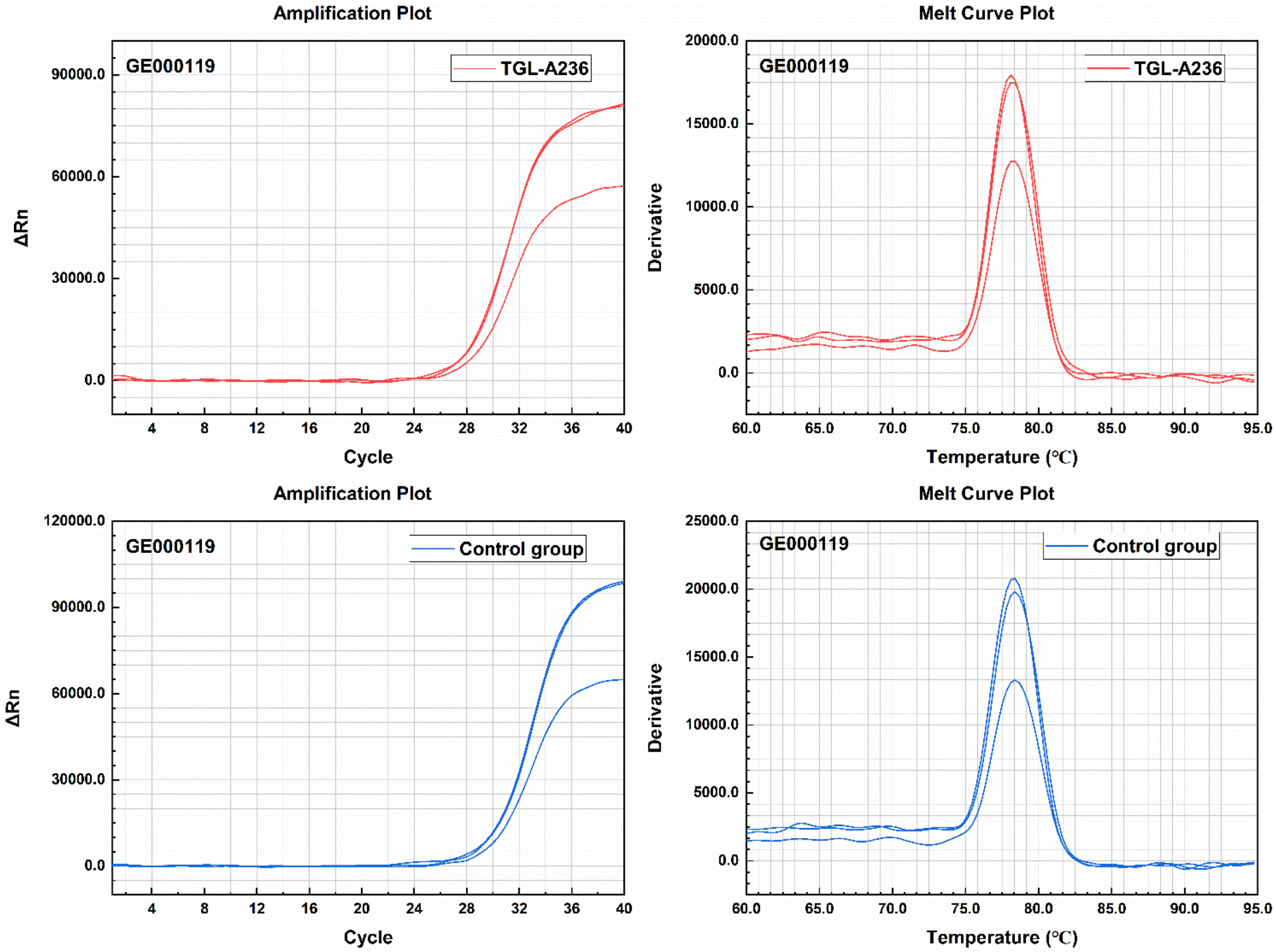

Supplementary Figure S3. Amplification and melt curves of the target gene *GE000119*. Red lines represent the mutant strain, blue lines represent the original strain.

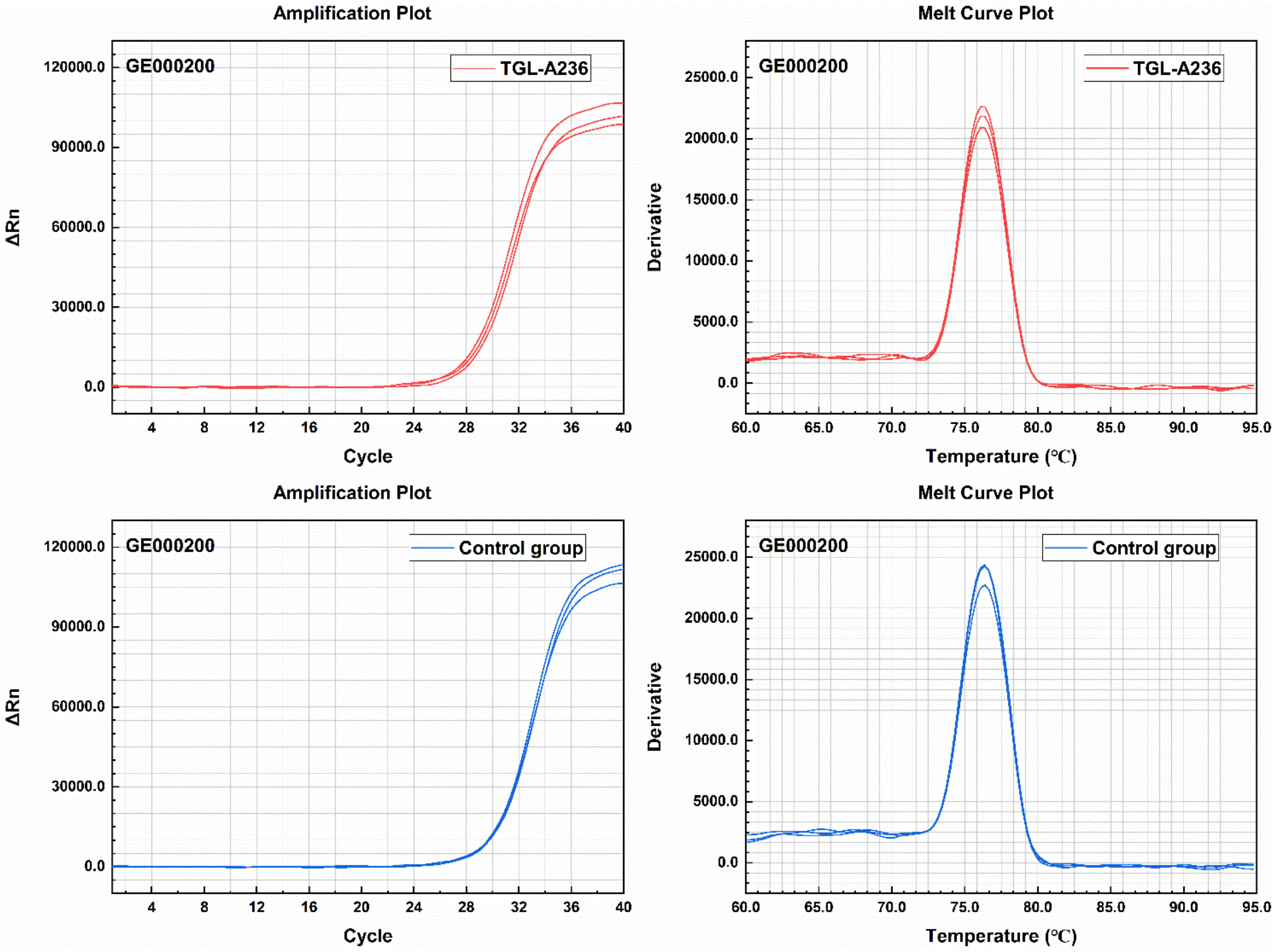

Supplementary Figure S4. Amplification and melt curves of the target gene *GE000200*. Red lines represent the mutant strain, blue lines represent the original strain.

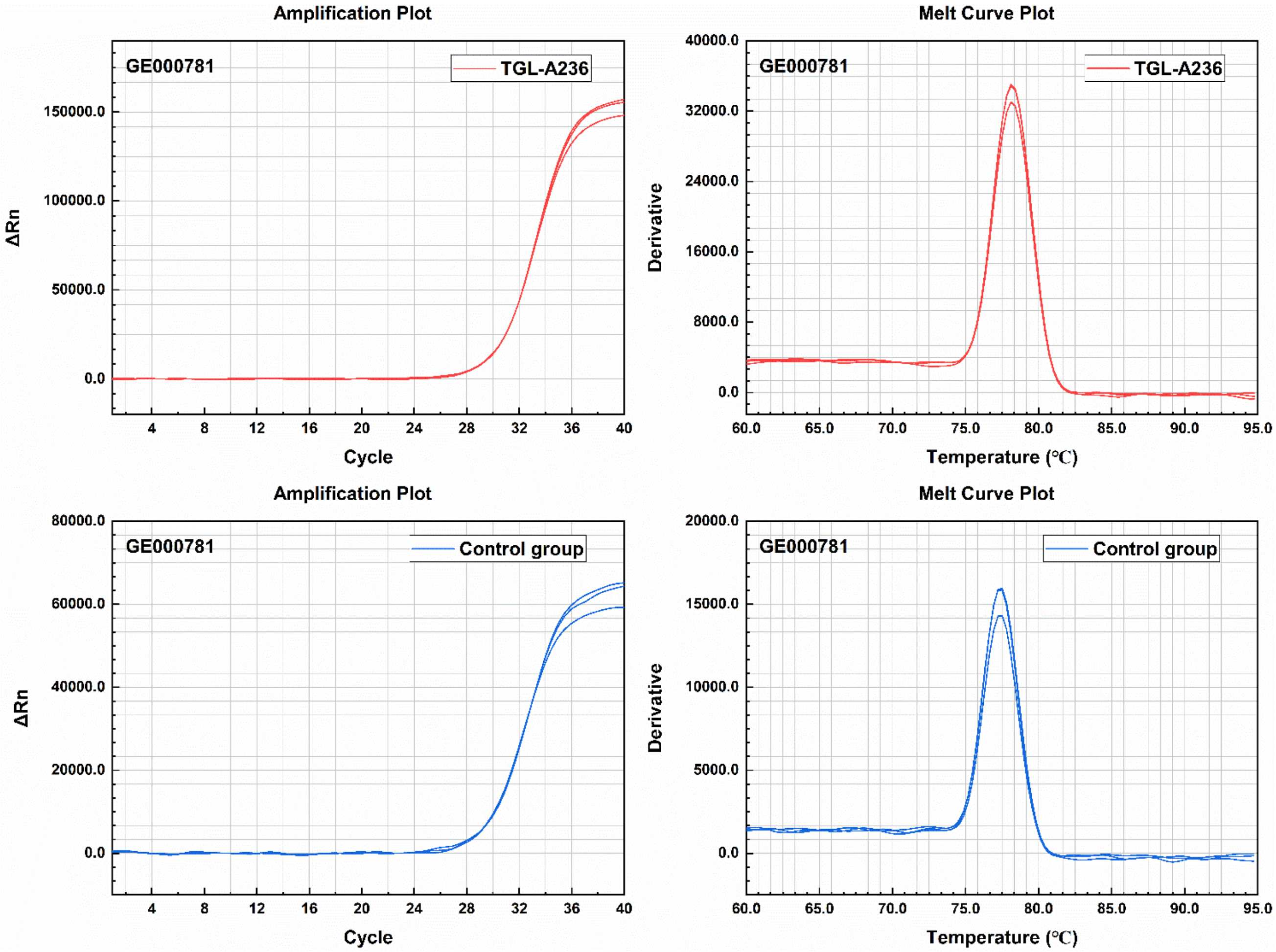

Supplementary Figure S5. Amplification and melt curves of the target gene *GE000781*. Red lines represent the mutant strain, blue lines represent the original strain.

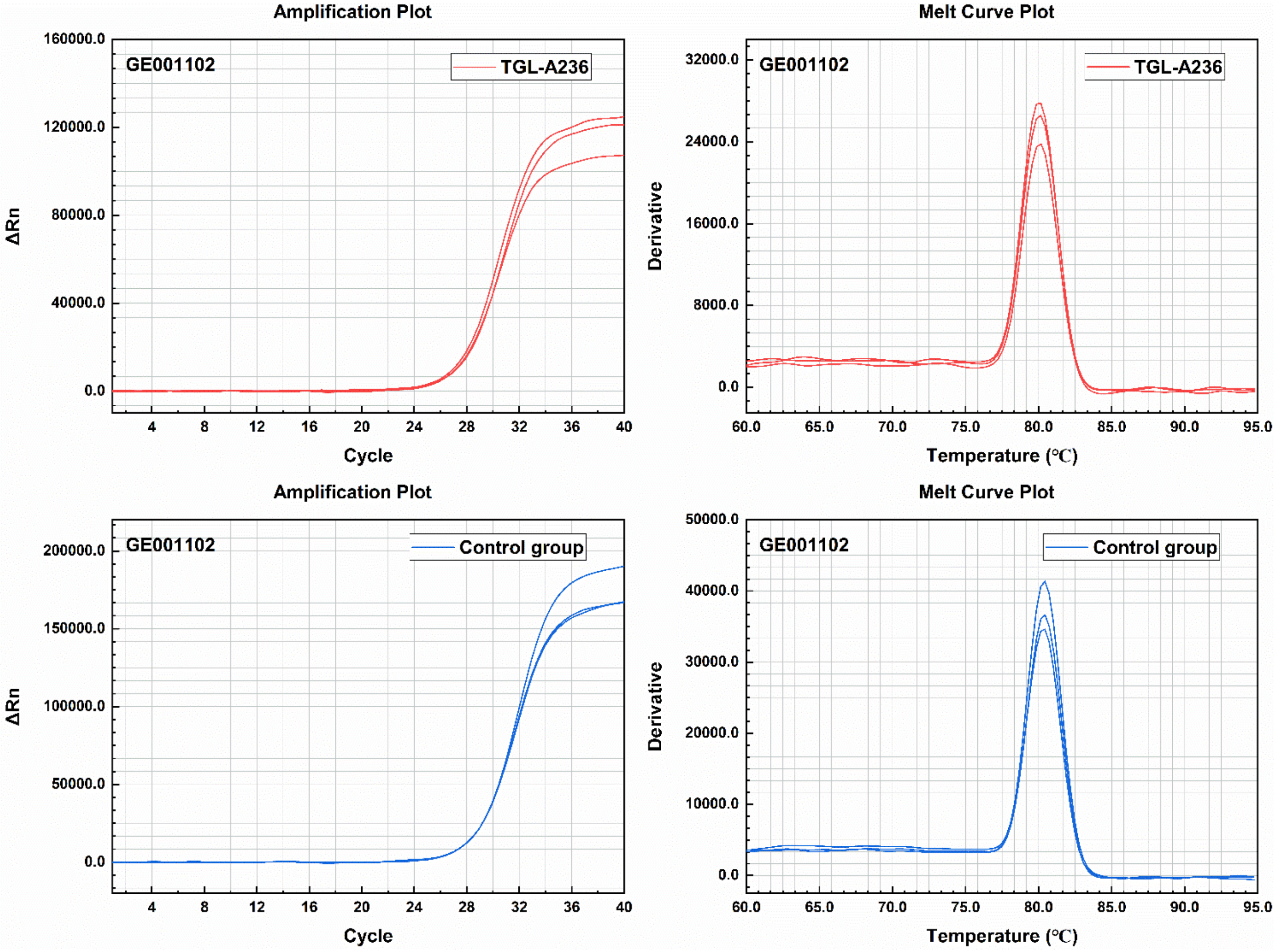

Supplementary Figure S6. Amplification and melt curves of the target gene *GE001102*. Red lines represent the mutant strain, blue lines represent the original strain.

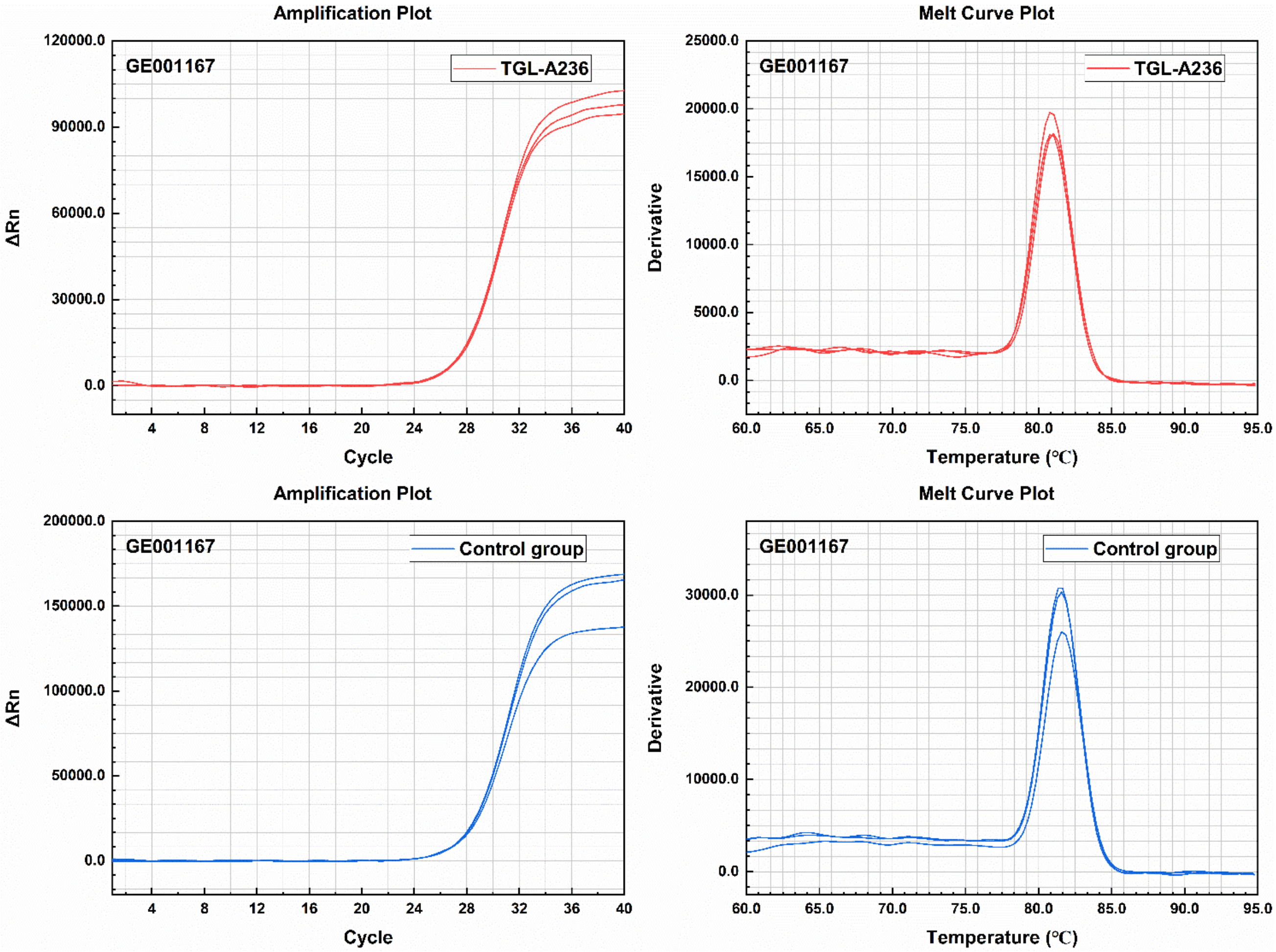

Supplementary Figure S7. Amplification and melt curves of the target gene *GE001167*. Red lines represent the mutant strain, blue lines represent the original strain.

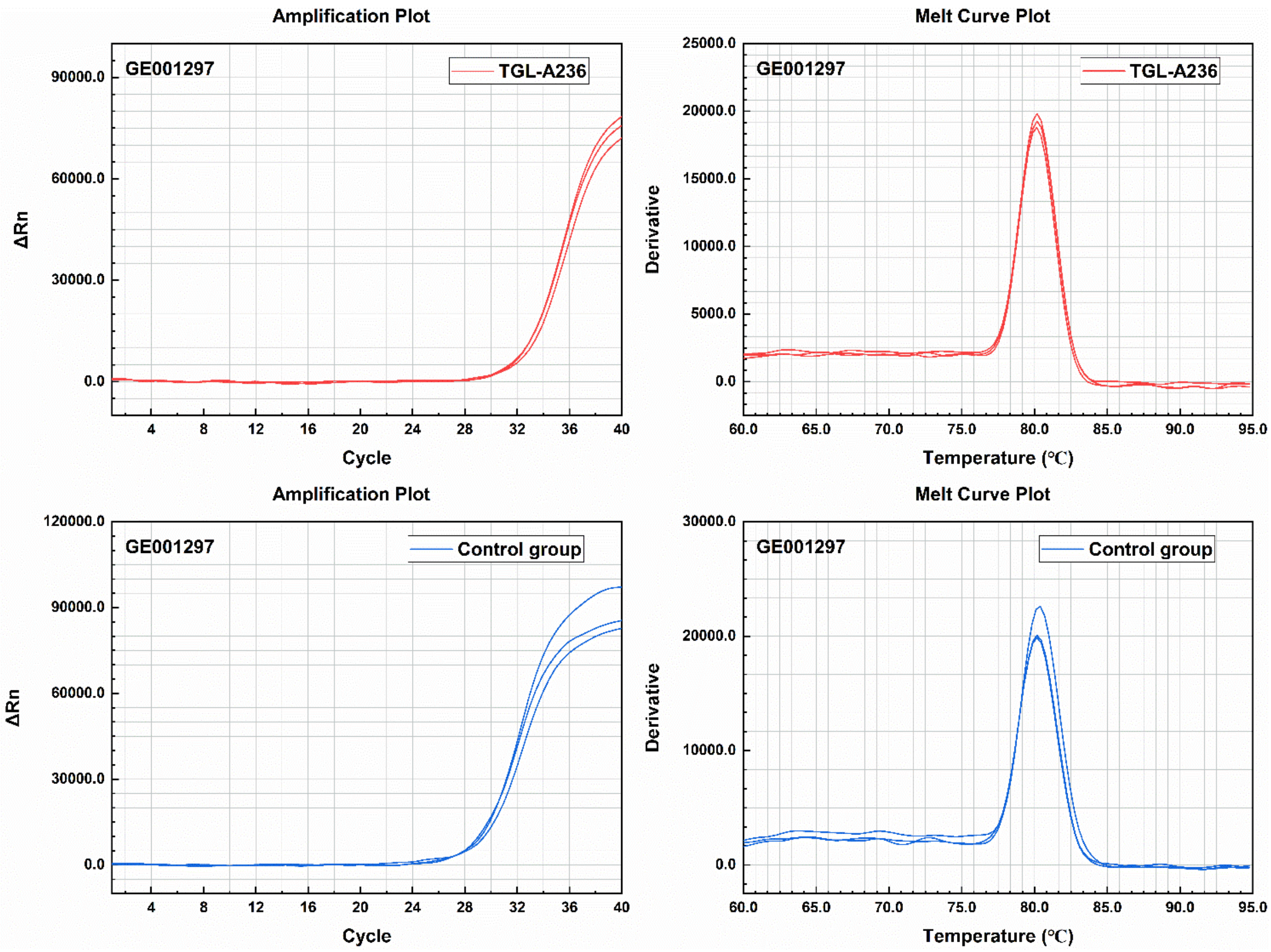

Supplementary Figure S8. Amplification and melt curves of the target gene *GE001297*. Red lines represent the mutant strain, blue lines represent the original strain.

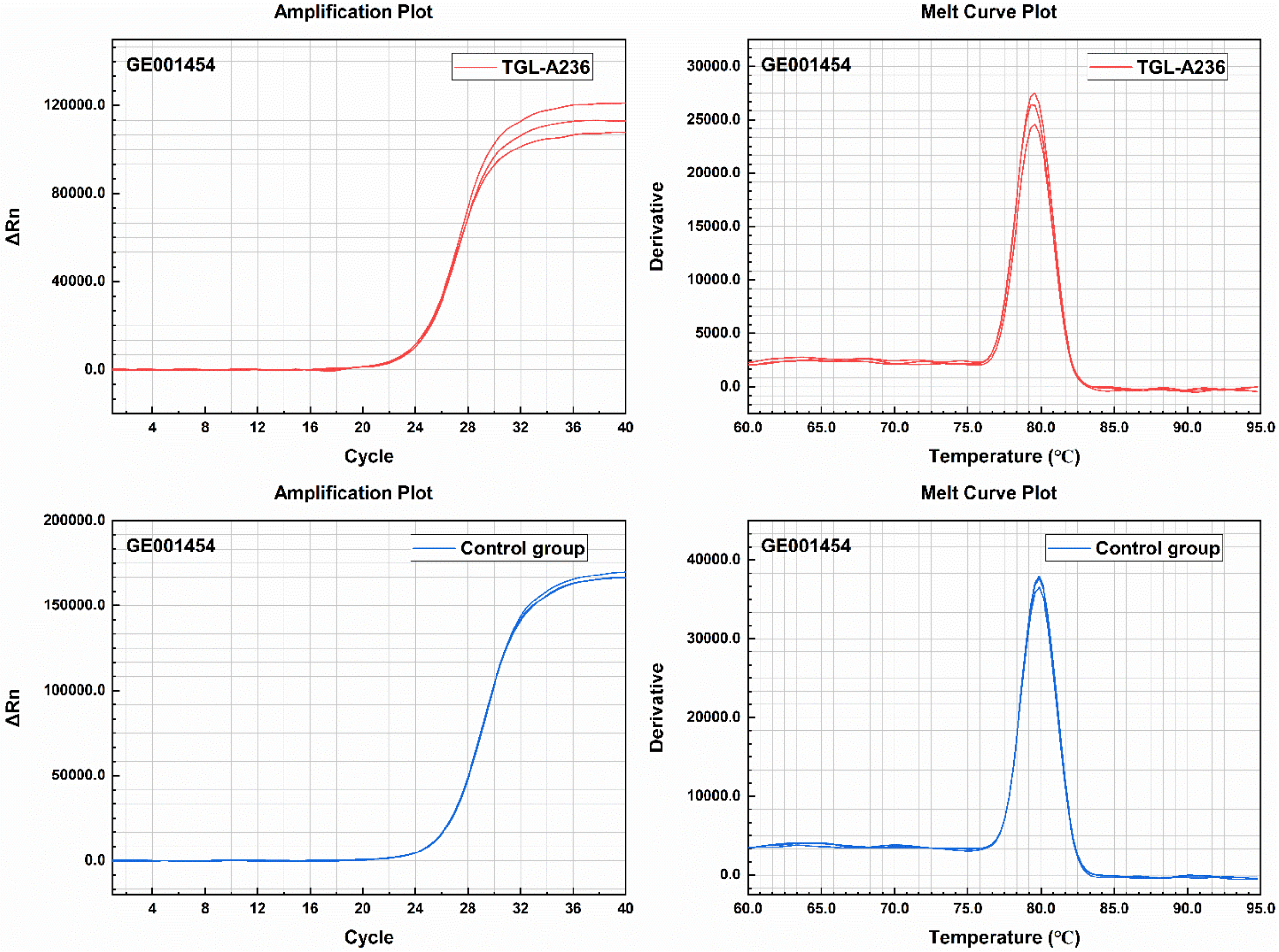

Supplementary Figure S9. Amplification and melt curves of the target gene *GE001454*. Red lines represent the mutant strain, blue lines represent the original strain.

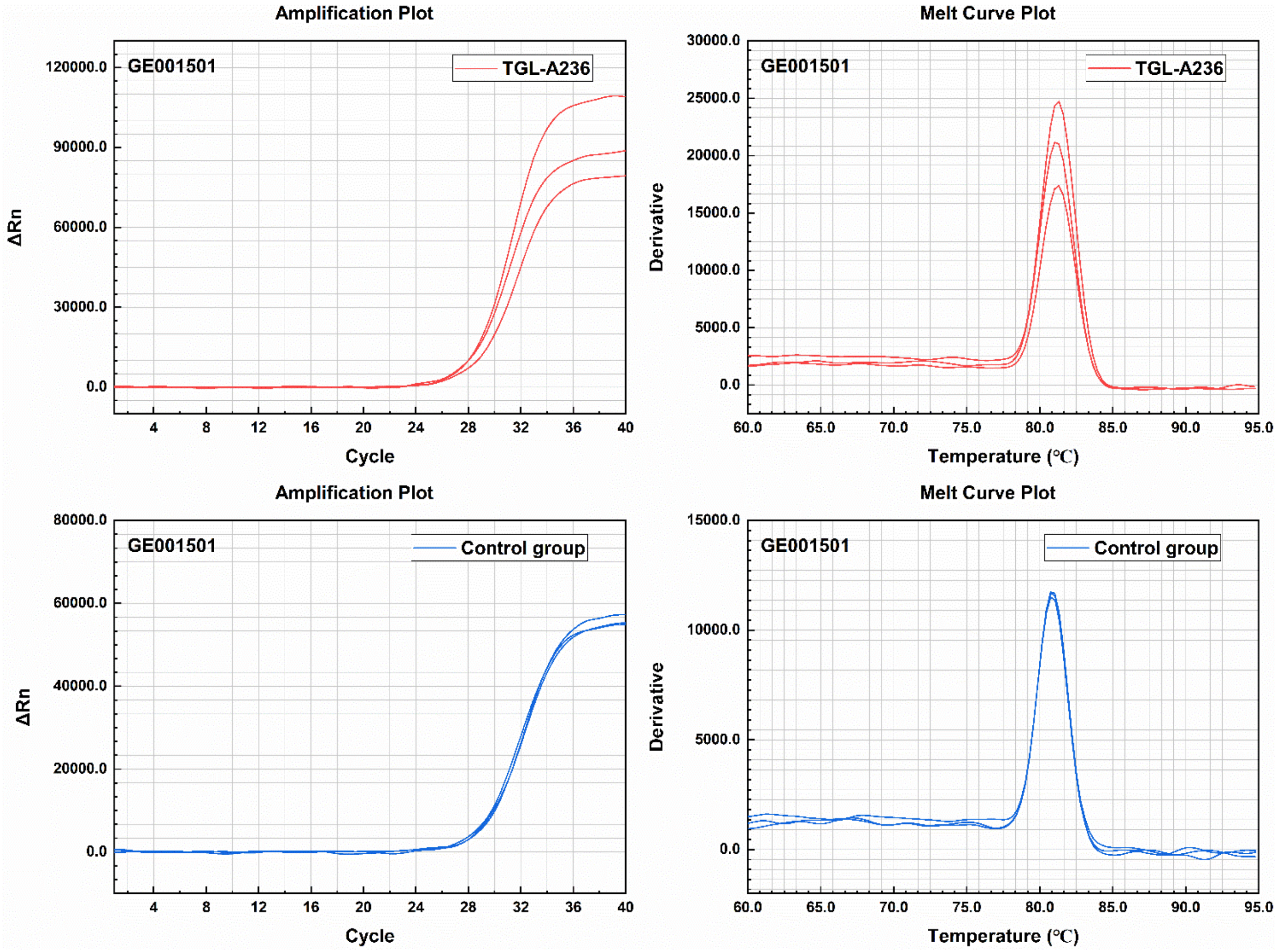

Supplementary Figure S10. Amplification and melt curves of the target gene *GE001501*. Red lines represent the mutant strain, blue lines represent the original strain.

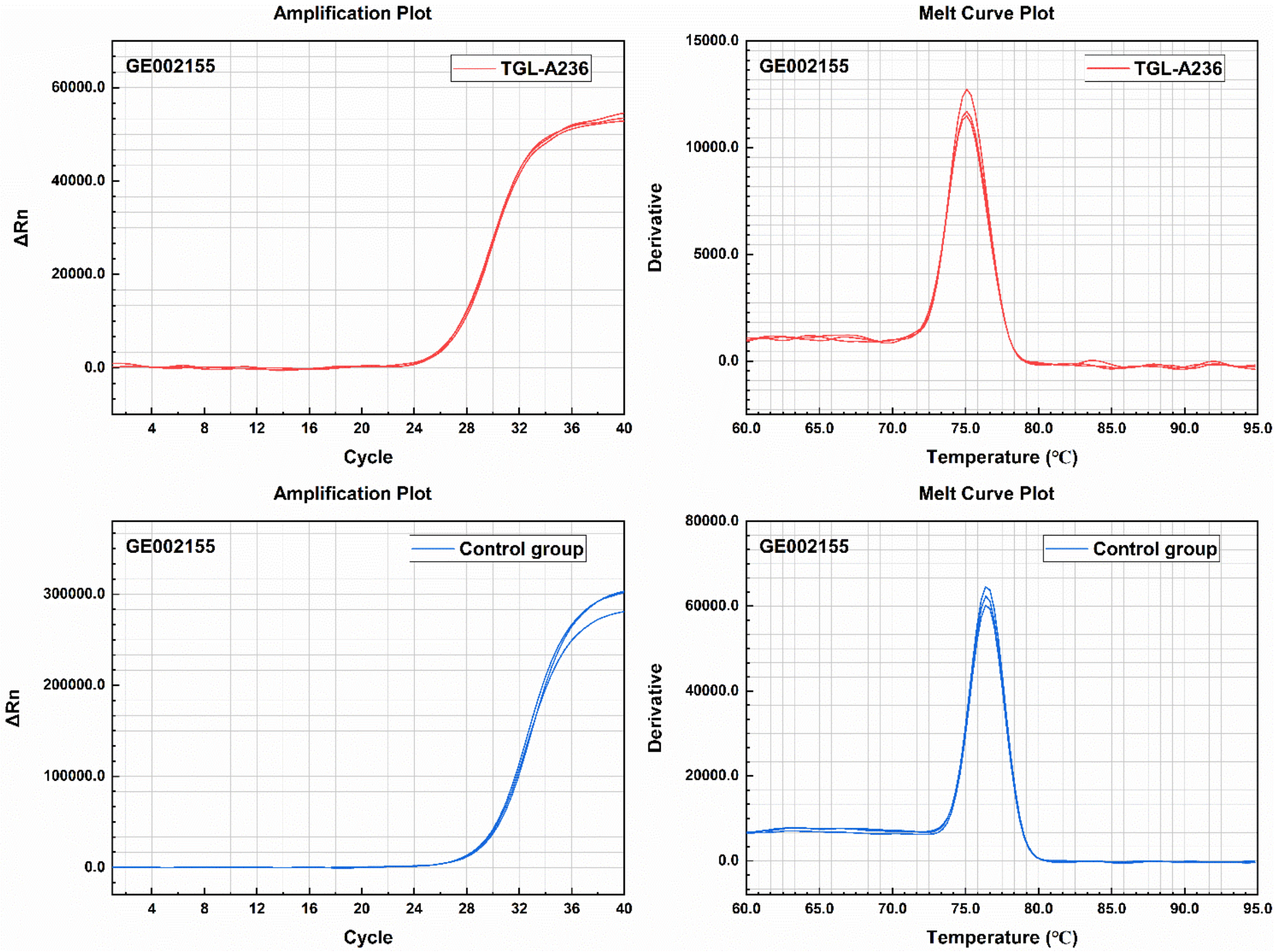

Supplementary Figure S11. Amplification and melt curves of the target gene *GE002155*. Red lines represent the mutant strain, blue lines represent the original strain.

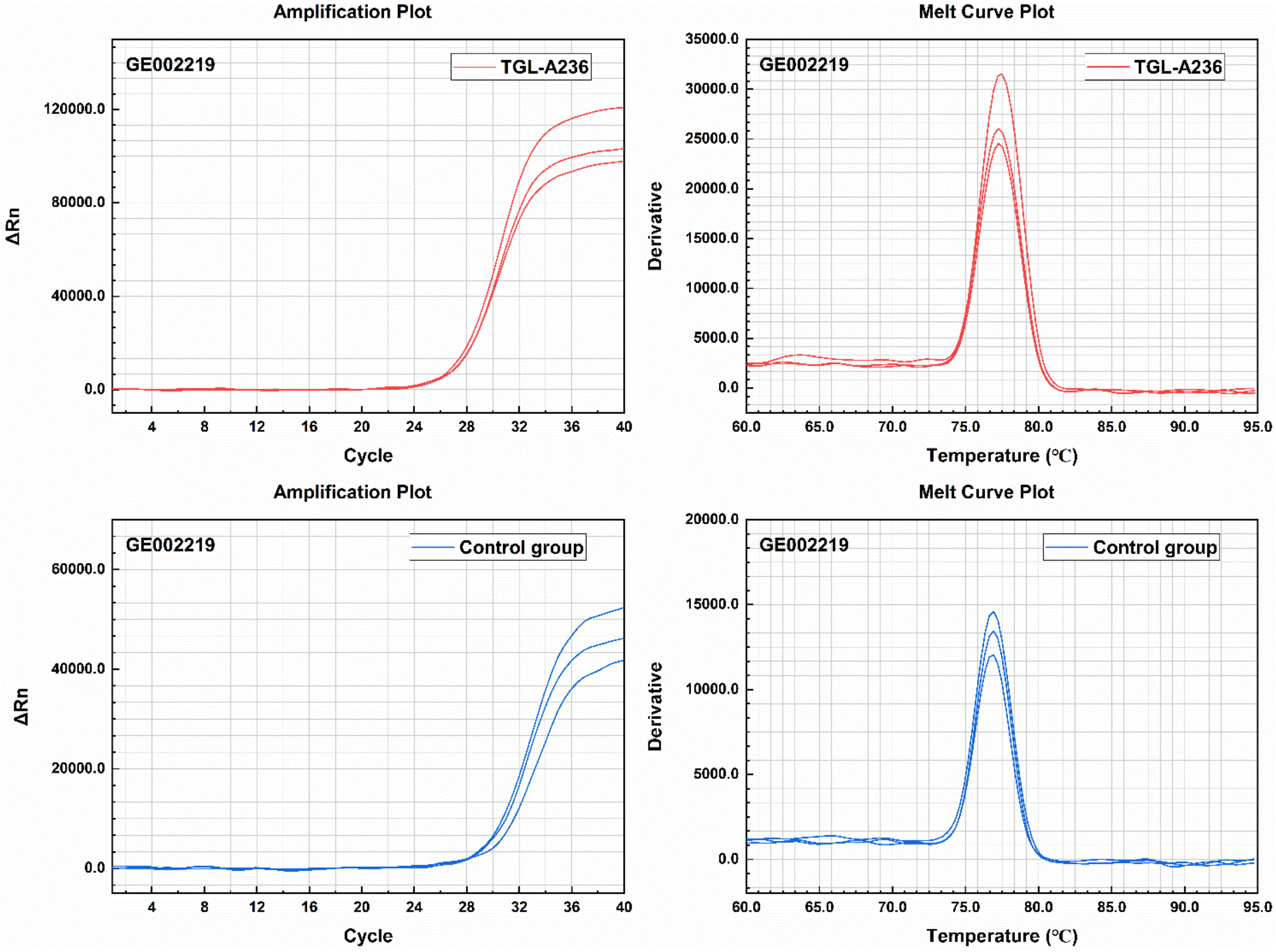

Supplementary Figure S12. Amplification and melt curves of the target gene *GE002219*. Red lines represent the mutant strain, blue lines represent the original strain.

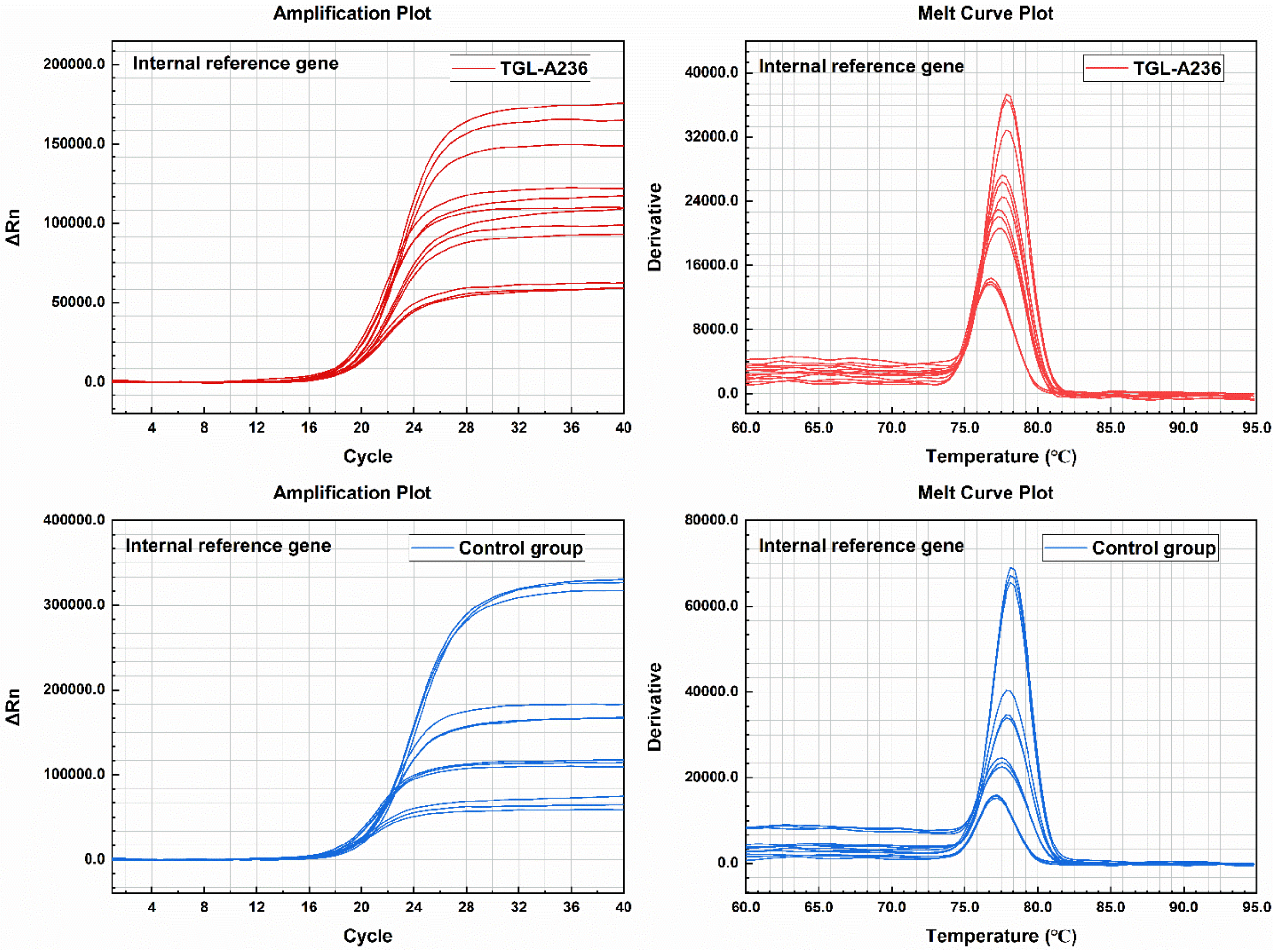

Supplementary Figure S13. Amplification and melt curves of the internal reference gene (*thl* gene). Red lines represent the mutant strain, blue lines represent the original strain.

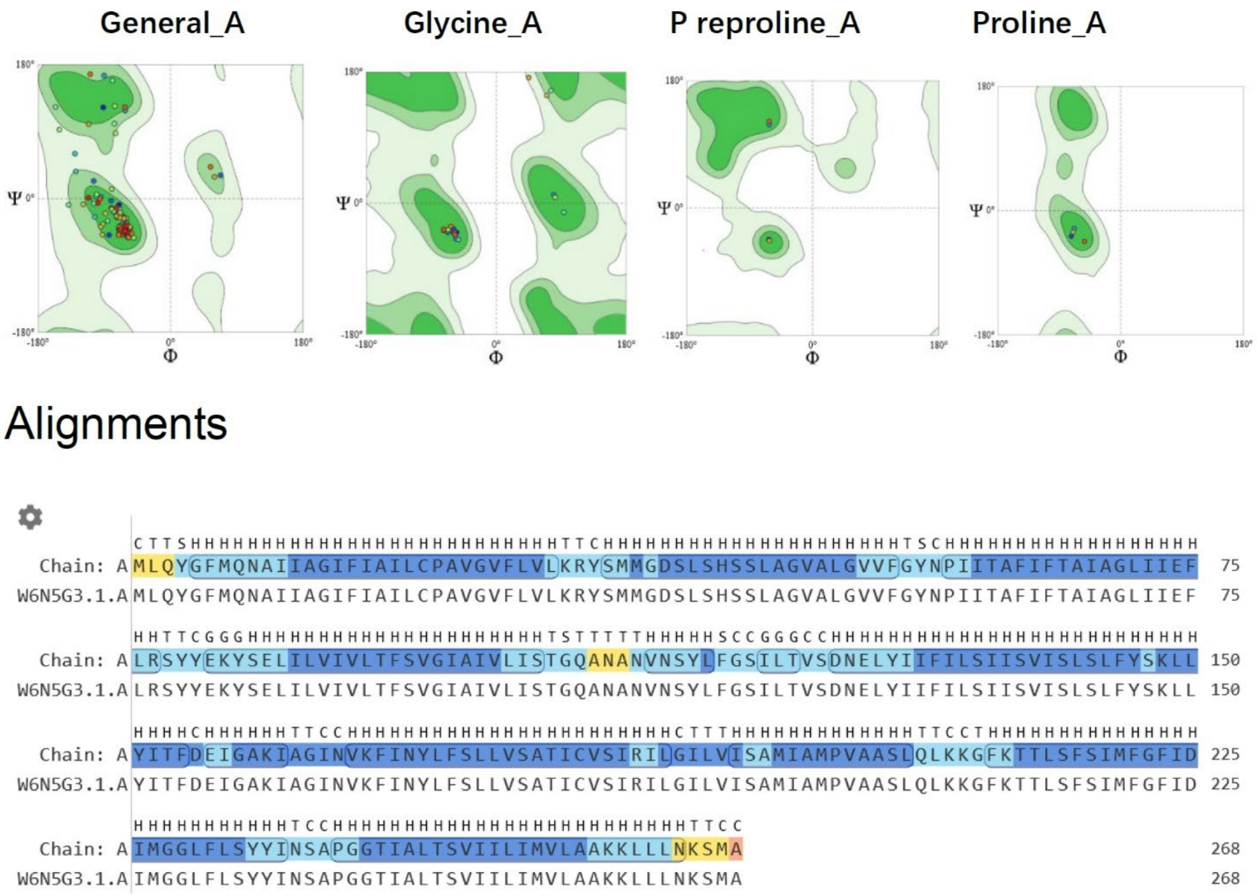

Supplementary Figure S14. General, Glycine, Pre-proline, Proline and Sequence Alignments for homology modeling of *GE000119* gene encoded protein.

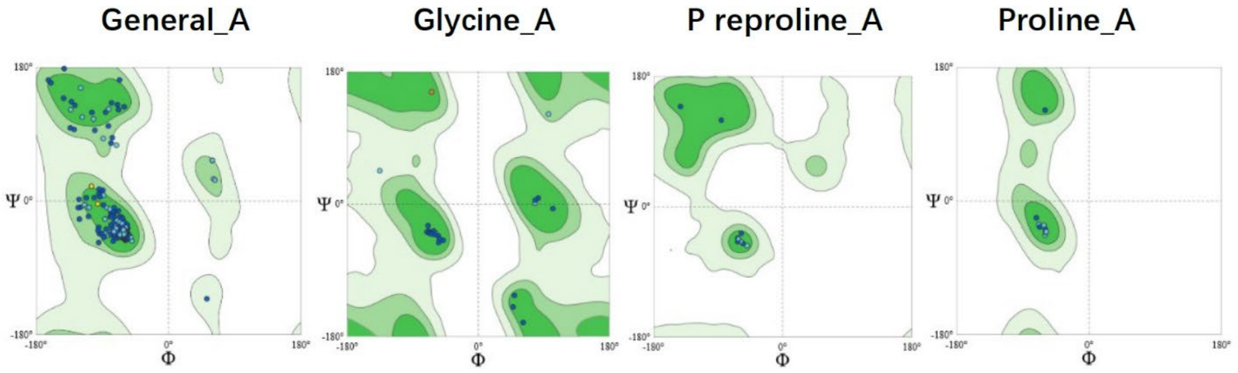

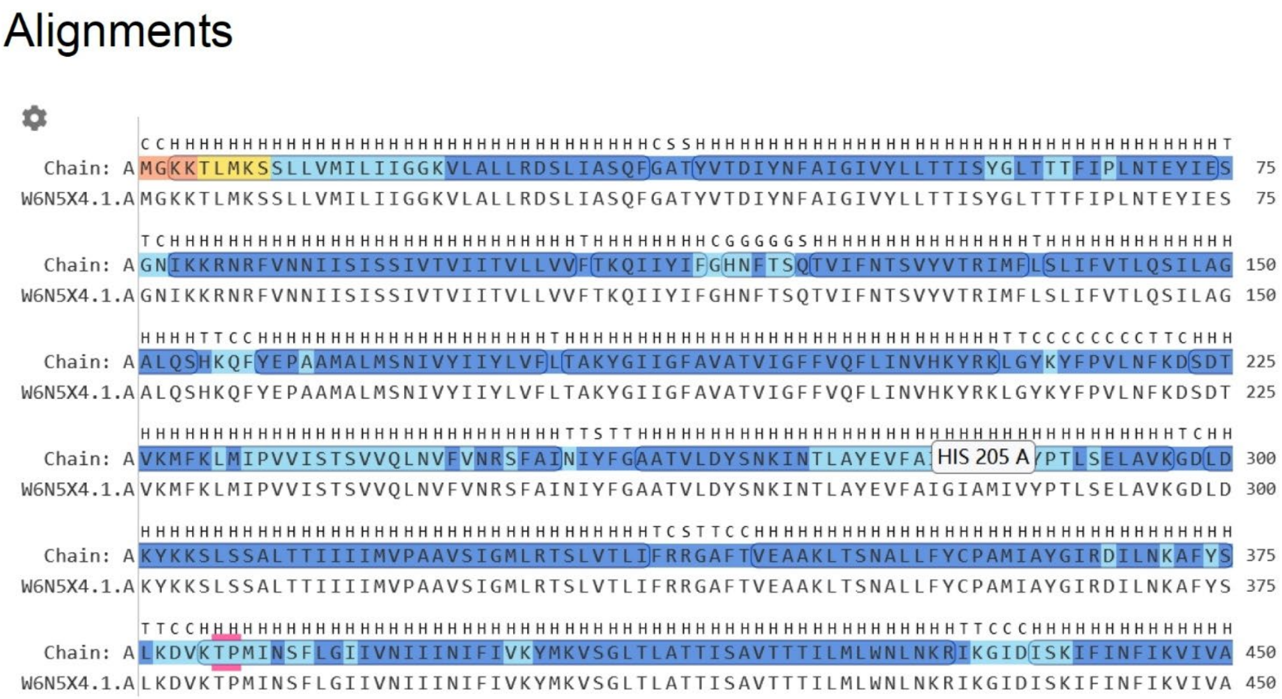

Supplementary Figure S15. General, Glycine, Pre-proline, Proline and Sequence Alignments for homology modeling of *GE000200* gene encoded protein.

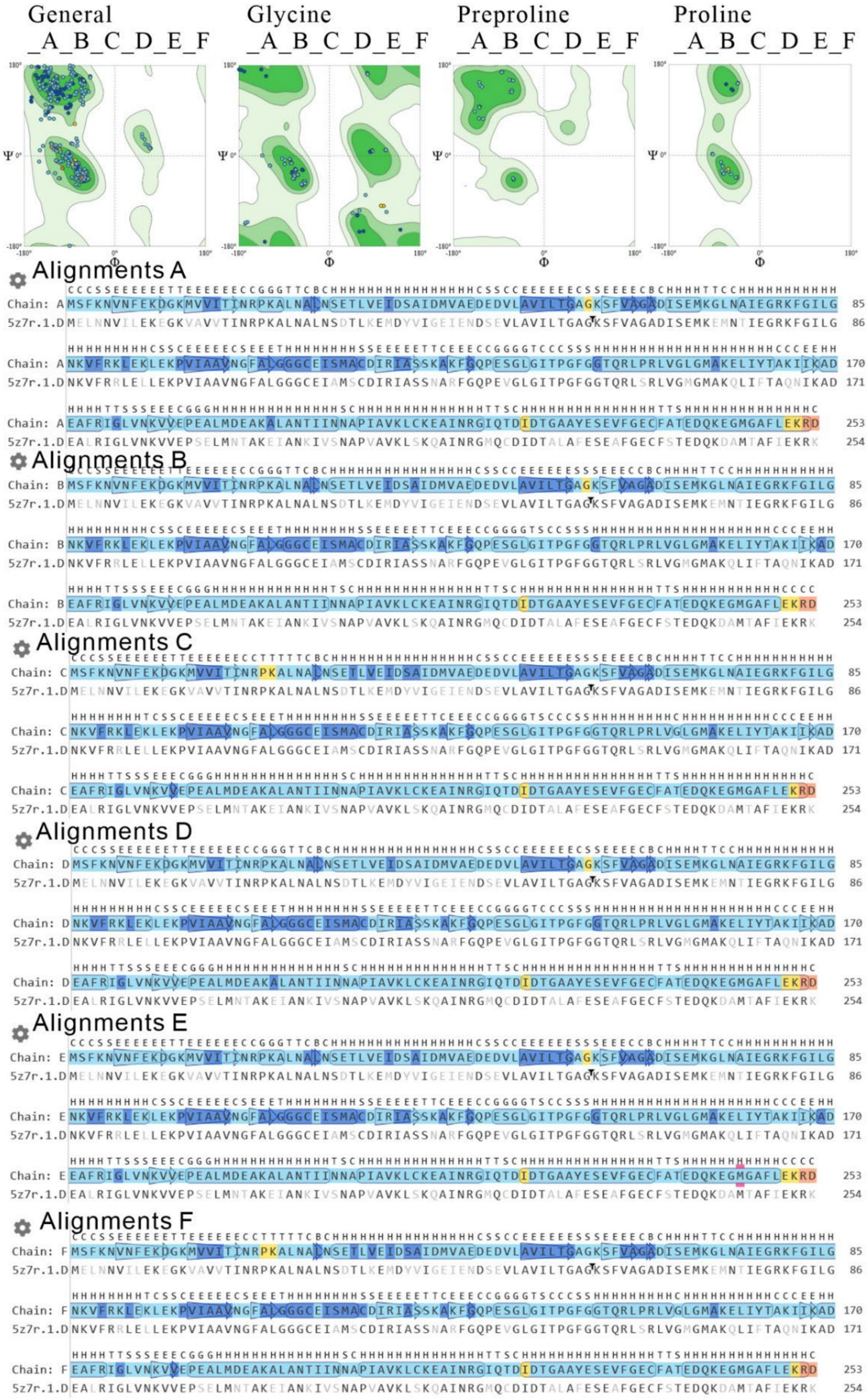

Supplementary Figure S16. General, Glycine, Pre-proline, Proline and Sequence Alignments for homology modeling of *GE001454* gene encoded protein.

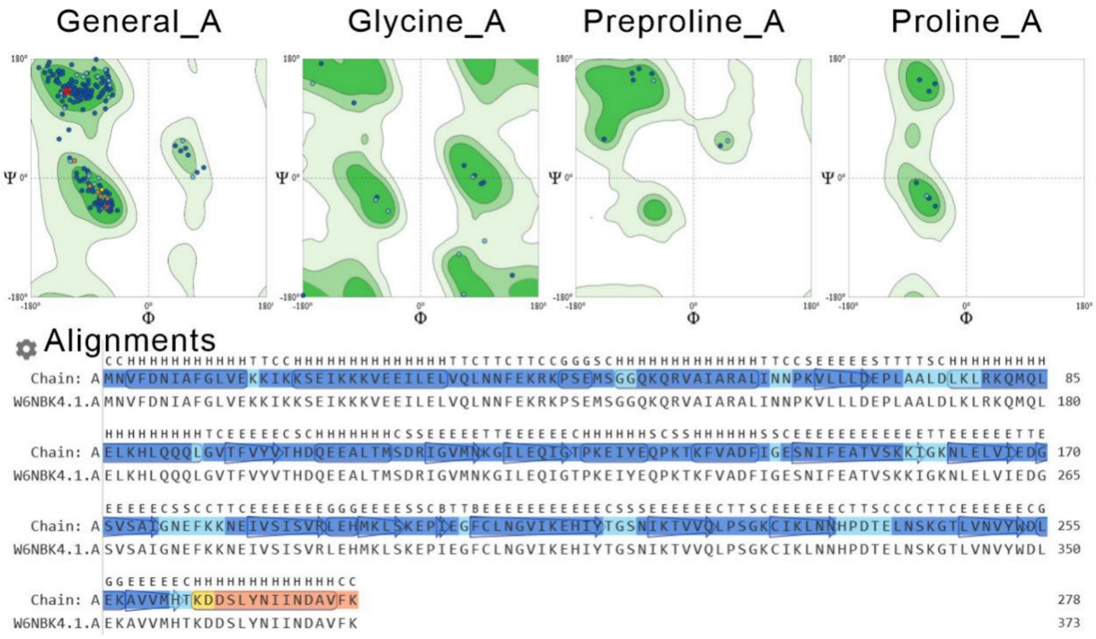

Supplementary Figure S17. General, Glycine, Pre-proline, Proline and Sequence Alignments for homology modeling of *GE001501* gene encoded protein.

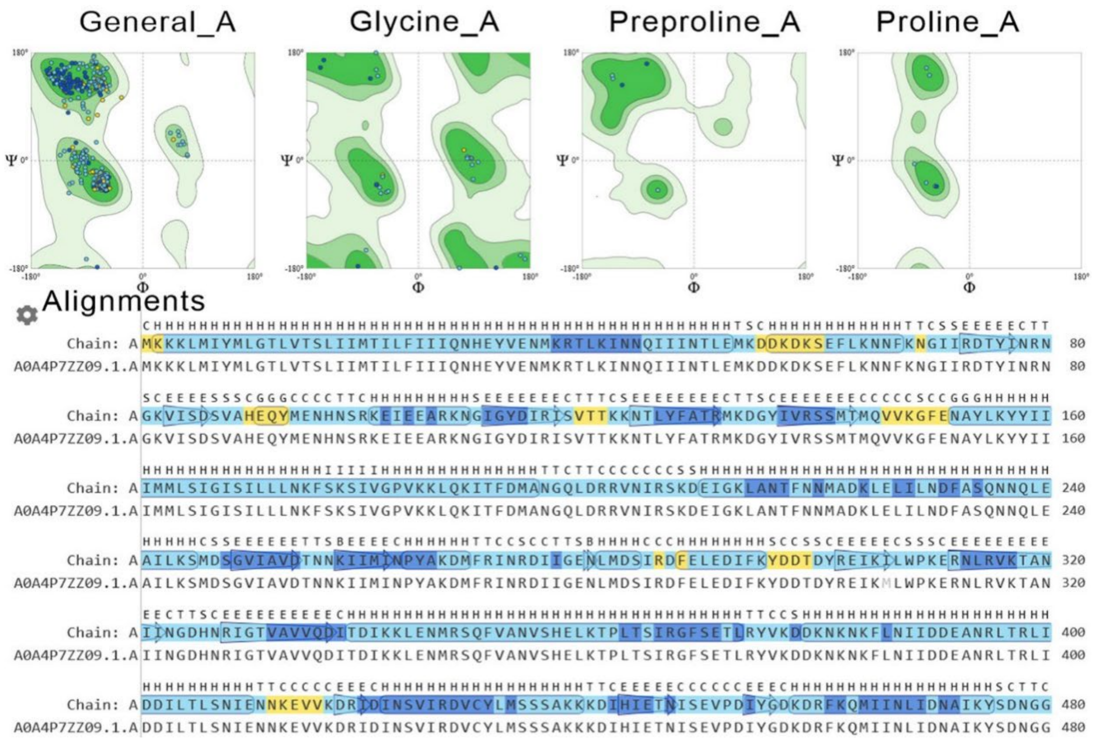

Supplementary Figure S18. General, Glycine, Pre-proline, Proline and Sequence Alignments for homology modeling of *GE002219* gene encoded protein.

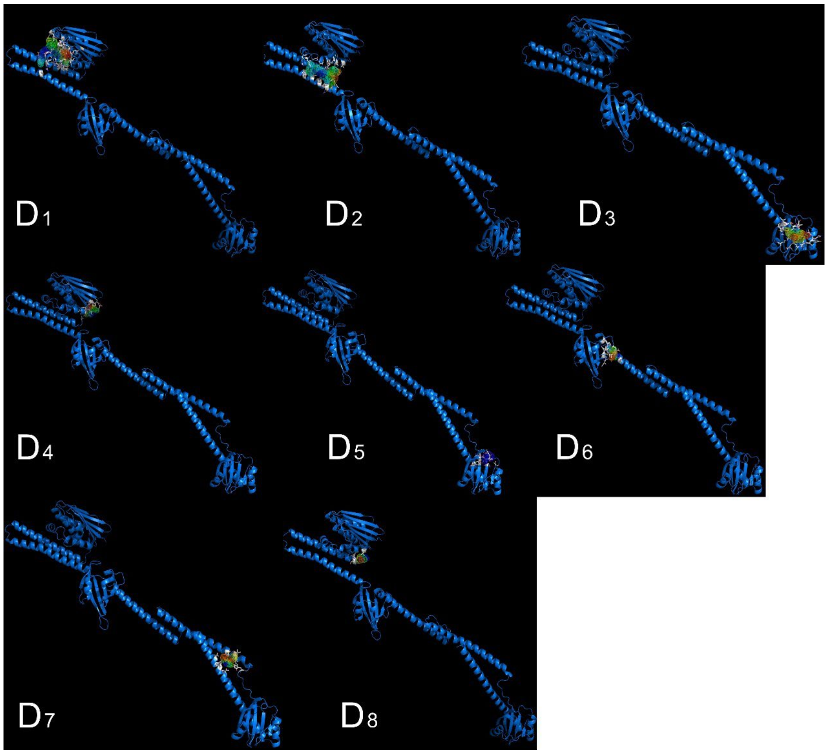

Supplementary Figure S19. The specific positions of the cavities are sorted by surface volume for homology modeling of *GE002219* gene encoded protein.

1.2 Supplementary Tables

Table S1. Comparison of the main toxic substances in SSJ before and after treatment.

Chemical name	Relative percentage of substance before treatment (%)	Relative percentage of substance after treatment (%)	CAS Number	SMILES	Molecular Mass (g/mol)	Estimated LogKow	Structure	References
1,2,3-Propanetricarboxylic acid, 2-hydroxy-	0.920%	-	77-92-9	O=C(O)C(O)(CC(=O)O) CC(=O)O	192.13	-1.6734	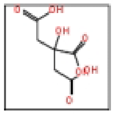	Takács-Novák et al., 1997
Dicarbonic acid, diethyl ester	1.254%	0.883%	1609-47-8	O=C(OC(=O)OCC)OCC	162.14	0.1258	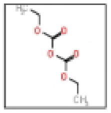	Meylan and Howard, 1995
2-Propenoic acid, 3-(2-hydroxyphenyl)-, (E)-	0.878%	0.481%	614-60-8	O=C(O)C=Cc(c(O) ccc1)c1	164.16	1.5905	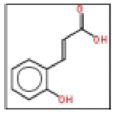	MEYLAN,WM & HOWARD,PH (1995)
Benzene, ethynyl-	1.158%	0.406%	536-74-3	C(c(cccc1)c1)#C	102.14	2.2598	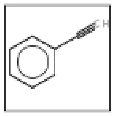	HANSCH,C ET AL. (1995)

L-Phenylalanine	0.385%	-	63-91-2	O=C(O)C(N)Cc(cccc1)c1	165.19	-1.2826	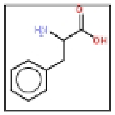	AVDEEF,A (1997)
1-Propene-1,2,3-tricarboxylic acid, (E)-	0.357%	0.161%	4023-65-8	O=C(O)C(=CC(=O)O)CC(=O)O	174.11	-0.1442	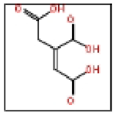	-
4-Pyridinecarboxylic acid, methyl ester	0.304%	0.204%	2459-09-8	O=C(OC)c(ccnc1)c1	137.14	0.6397	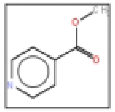	HANSCH,C ET AL. (1995)
1H-Purin-6-amine	0.211%	0.028%	73-24-5	n(c(c(ncn1)c1n2)N)c2	153.13	-0.7259	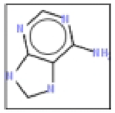	HANSCH,C ET AL. (1995)
1H-Indole	0.090%	0.046%	120-72-9	C1ccc2cc[nH]c2c1	117.15	2.0548	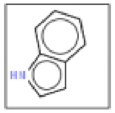	YALKOWSKY,SH &DANNENFELSER,RM (1992)
Benzaldehyde	0.050%	0.017%	100-52-7	O=Cc(cccc1)c1	106.13	1.7102	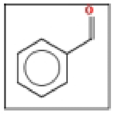	YALKOWSKY,SH & HE,Y (2003)
2-Propenoic acid, 3-(2-hydroxyphenyl)-, (E)-	0.047%	0.021%	614-60-8	O=C(O)C=Cc(c(O)ccc1)c1	164.16	1.5905	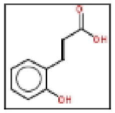	MEYLAN,WM ET AL. (1996)

Citrinin	0.034%	-	518-75-2	CC2OC=C1C(=C(C(O)=O)C(=O)C(=C1C2C)C)O	250.25	0.4516	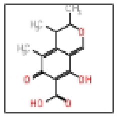	MEYLAN,WM & HOWARD,PH (1995)
2H-1,4-Benzodiazepin-2-one, 7-chloro-1,3-dihydro-3-hydroxy -1-methyl-5-phenyl-	0.016%	0.004%	846-50-4	CN2C(=O)C(O)N=C(c1ccccc1)c3cc(Cl)ccc23	300.75	2.1496	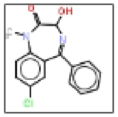	HANSCH,C ET AL. (1995)
1-Dodecanamine, N,N-dimethyl-, N-oxide	0.031%	0.015%	1643-20-5	O=N(CCCCCCCCCCCC)(C)C	229.41	4.673	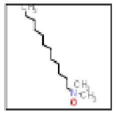	BROWN,SL ET AL. (1975C)
Imidazol-1-yl-acetic acid	0.005%	-	22884-10-2	N1(CC(=O)O)C=NC=C1	126.12	-0.8898	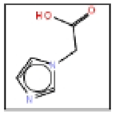	-
3-PYRIDINOL, 2-METHYL-	0.012%	-	1121-25-1	n(ccc1)c(c1O)C	109.13	0.8716	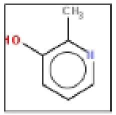	-
N-Benzylformamide	0.296%	0.184%	6343-54-0	C(=O)NCc1ccccc1	135.17	0.5629	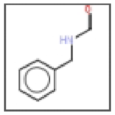	-
2,4-Hexadienoic acid,	0.067%	0.045%	110-44-1	O=C(O)C=CC=CC	112.13	1.6212	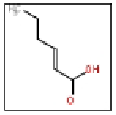	NEELY,WB & BLAU,GE (1985)

(E,E)-								
Benzaldehyde, 4-hydroxy-3-methoxy-	0.007%	0.004%	121-33-5	O=Cc(ccc(O)c1OC)c1	152.15	1.0549	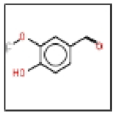	YAWS,CL (1994B)

"-" indicates that the relative percentage of the substance is zero or relevant references cannot be consulted.

References

Blau, G. E., and Neely, W. B. (1985). Environmental exposure from chemicals: CRC Press.

Brown, S., Chan, F., Jones, J., Liu, D., and McCaleb, K. (1975). Research Program on Hazard Priority Ranking of Manufactured Chemicals. Phase II.(Chemical 61-79).

Hansch, C., Hoekman, D., Leo, A., Zhang, L., and Li, P. (1995). The expanding role of quantitative structure-activity relationships (QSAR) in toxicology. *Toxicol. Lett.* 79 (1-3):45-53. doi: 10.1016/0378-4274(95)03356-P.

Meylan, W. M., and Howard, P. H. (1995). Atom/fragment contribution method for estimating octanol–water partition coefficients. *J. Pharm. Sci.* 84 (1):83-92. doi: 10.1002/jps.2600840120.

Meylan, W. M., Howard, P. H., and Boethling, R. S. (1996). Improved method for estimating water solubility from octanol/water partition coefficient. *Environ. Toxicol. Chem.* 15 (2):100-106. doi: 10.1002/etc.5620150205.

Takács-Novák, K., Box, K. J., and Avdeef, A. (1997). Potentiometric pKa determination of water-insoluble compounds: validation study in methanol/water mixtures. *Int. J. Pharm.* 151 (2):235-248. doi: 10.1016/S0378-5173(97)04907-7.

Yalkowsky, S., and He, Y. (2003). Handbook of Aqueous Solubility Data CRC, Boca Raton, FL.

Yalkowsky, S. H., and Dannenfelser, R. M. (1992). Aquasol database of aqueous solubility. *College of Pharmacy, University of Arizona, Tucson, AZ.* 189.

Yaws, C. (1994). Handbook of Vapor Pressure Vol. 3: C8–C28 Compounds. *Houston, TX: Gulf Pub. Co.*
